# IL-8 receptor signaling as a novel target for angiogenic retinopathies

**DOI:** 10.1007/s10456-025-10015-7

**Published:** 2025-11-01

**Authors:** Maximilian J. Garcia, Amanda L. Beall, Monica S. Morales, Nolan J. Beatty, Samuel A. Palmer, Marvarakumari Jhala, Aleksandra Drmanovic, Stephen Priest, Yueli Zhang, Rong Yang, Kyana Arellano, John S. Penn, Dolly A. Padovani-Claudio

**Affiliations:** 1 Department of Ophthalmology and Visual Sciences, Vanderbilt University Medical Center, 2311 Pierce Avenue, Nashville, TN 37232 USA; 2 Vanderbilt University School of Medicine, Nashville, TN USA

**Keywords:** CXCR2, IL-8, Retinopathy, Angiogenesis, Human Müller cells, Chemokines

## Abstract

Diabetic retinopathy (DR) is characterized by chronic retinal inflammation and vascular remodeling that can threaten vision. Most current treatments are administered intravitreally and target vascular endothelial growth factor A (VEGF) but are often ineffective. Nevertheless, few alternative treatments, and no oral DR therapies, exist. Although IL-1β, TNFα, and IL-8 are upregulated along with VEGF within eyes with DR, they are not therapeutically targeted. IL-8 levels correlate with DR progression and resistance to anti-VEGF therapy, suggesting VEGF-independent contributions of IL-8-receptor signaling to DR. IL-1β and TNFα, in turn, enhance expression of pro-angiogenic CXCR2 ligands (e.g. IL-8, CXCL1) in human Müller cells (hMC). Despite investigation of CXCR2 roles in several angiogenic and fibrotic diseases, CXCR2 inhibitors have not been explored in DR models. In this study, we show protein upregulation of IL-8 and CXCL1, but no detectable VEGF in conditioned media (CM) from IL-1β and TNFα-stimulated hMC. Stimulation of human retinal microvascular endothelial cells (hRMEC) with this human Müller cell-conditioned media (hMC-CM), as well as directly with IL-8, upregulated hRMEC proliferation and migration. CXCR2 inhibition reduced pro-angiogenic hRMEC responses to hMC-CM and IL-8. Likewise, in vivo, in the oxygen-induced retinopathy (OIR) model, either genetic (*Cxcr2*^*-/-*^) or pharmacologic (SB225002) CXCR2 inhibition reduced pre-retinal neovascularization without altering avascularity or VEGF expression. These findings suggest that: (a) Müller cells may link inflammatory and angiogenic responses in the retina, (b) CXCR2 activation may contribute to DR, and (c) CXCR2 inhibitors may be repurposed to reduce pre-retinal neovascularization, a key feature of proliferative DR.

## Introduction

Diabetic retinopathy (DR) is a potentially blinding complication of diabetes mellitus characterized by chronic inflammation and vascular remodeling in the retina [1]. It remains a leading cause of blindness in working-age adults, and its worldwide prevalence is expected to increase by nearly 60% from 2020 to 2045 [2]. The repurposing of vascular endothelial growth factor A (VEGF) inhibitors, originally formulated for cancer therapy, changed DR therapy outcomes from vision preservation to vision improvement. However, a significant proportion of patients are refractory to anti-VEGF therapies, leading to treatment failure [3]. In addition, there are no systemically formulated therapies for DR, and anti-VEGF drugs must be periodically injected into the eyes. This underscores the need for novel therapeutic approaches for DR management.

Various cytokines (IL-1β, TNFα), chemokines (IL-8), and growth factors (VEGF) have been found to accumulate in the eyes in patients with DR [4, 5]. These are upregulated by hyperglycemia, hyperlipidemia, and chronic systemic inflammation, which are known risk factors for the development and progression of DR [6]. Intraocular VEGF levels in patients with diabetes are highly correlated with progression to proliferative DR (PDR), making VEGF an obvious drug target for intraocular DR drugs. However, IL-8, IL-1β, and TNFα are not only elevated along with VEGF in the vitreous of patients with DR, but also systemically [5, 7]. Specifically, increased vitreous and serum levels of IL-8, a potent neutrophil chemoattractant and pro-angiogenic chemokine, correlate with progression to PDR *and* development of diabetic macular edema (DME) [4, 5, 7], both blinding features of DR. Furthermore, aqueous IL-8 levels inversely correlate with response to anti-VEGF therapy in DR [8]. Finally, polymorphisms in the promoter regions of CXCL8, the gene that encodes IL-8, are associated with DR susceptibility, as well as with susceptibility to other angiogenic conditions such as cancer and age-related macular degeneration (AMD) [9–12]. Likewise, SNPs in loci linked to genes encoding CXCR1 and CXCR2, the IL-8 receptors, are associated with angiogenic disease susceptibility, morbidity, and response to anti-VEGF therapies [10, 13, 14].

The IL-8:CXCR1/2 signaling system has been implicated in the induction of leukocyte chemotaxis, leukostasis, neutrophil degranulation, extracellular matrix remodeling, angiogenesis, and gliosis—all critical pathologic events in DR progression [9, 12, 15–21]. Furthermore, IL-8 and/or CXCR2 are expressed by leukocytes, astrocytes, pericytes, microglia, retinal microvascular endothelial cells (RMEC), and Müller cells (MC)—key players in DR pathogenesis, implying that IL-8:CXCR2 signaling could modulate retinal pathology [22, 23]. Yet, CXCR2 inhibitors, many of which are in clinical trials for non-ocular angiogenic and inflammatory diseases, have not been explored as DR therapies, either intravitreally or systemically [24–26].

Our previous work has shown that stimulation of human Müller cells (hMC) with free fatty acids (mimicking hyperlipidemia) or IL-1β and TNFα (mimicking chronic inflammation) significantly upregulates hMC expression of several CXCR2 ligands, including IL-8 [27]. Similar results have been shown in MIO-M1 transformed Müller glia [28]. In addition, in mice, Müller cells have been shown to upregulate expression of the mouse IL-8 homologue, *Cxcl1*, during the time of peak neovascularization (NV) in the oxygen-induced retinopathy model (OIR), a rodent model of pre-retinal NV used to mimic PDR pathology [19].

Given that DR is a response to dysregulated retinal homeostasis and that MC are key players in mediating this homeostasis [29], we sought to explore: (1) how proliferation and migration of hRMEC in vitro are affected by paracrine effects of conditioned media from IL-1β/TNFα-stimulated hMC or after direct stimulation with IL-8; (2) the potential of CXCR1/2 inhibitors to alter these hRMEC responses; and (3) if and how interfering with CXCR2 signaling, either genetically or pharmacologically through intravitreal or systemic delivery, could affect pathogenic pre-retinal NV in the mouse OIR.

Our work demonstrates elevated levels of IL-8 and CXCL1 (but not VEGF) in the conditioned media from IL-1β and TNFα-stimulated hMC (hMC-CM). This conditioned media enhanced hRMEC proliferation and migration, which were dampened by CXCR2 inhibitors. We also show significant reduction in pre-retinal NV in OIR in CXCR2 knockout (*Cxcr2*^*-/-*^) mice, despite unaltered retinal expression levels of *Vegf* or its receptor. Finally, we found that either systemic or intravitreal CXCR2 pharmacologic inhibition reduced pre-retinal NV in OIR, at least as much as in *Cxcr2*^*-/-*^ mice. Our findings implicate CXCR2 activation in angiogenic phenotypes associated with DR progression to PDR and suggest that, if deemed safe for long term use in humans [30], CXCR2 inhibitors may help reduce retinal angiogenesis, even when administered systemically.

## Results

### Conditioned media from human Müller cells (hMC) stimulates human retinal microvascular endothelial cell (hRMEC) migration and proliferation

In our prior experiments with hMC, CXCR2 ligand transcripts ranked among the highest upregulated after stimulation with diabetes-relevant stimuli [27, 28]. In particular, *CXCL8* was among the top 10 differentially expressed genes (DEGs) after hMC stimulation with either IL-1β or TNFα, and *CXCL1* was among the top 10 after hMC stimulation with IL-1β [28] (Table 1). In these studies, genes encoding other CXCR2 ligands, including CXCL3 and CXCL5 were also within the top ten induced genes (not shown) [27, 28].

**Table 1:**
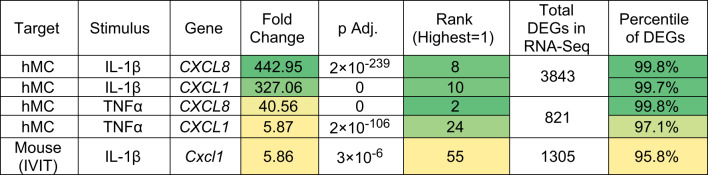
Induction of CXCR1/2 ligands after human Müller cell stimulation with IL-1β (1 ng/mL) or TNFα (1 ng/mL) or after mouse intravitreal injection of IL-1β (50 ng/mL)

Gene ranking (#1 = largest fold increase of ALL genes analyzed) reveals a potent induction in hMC of genes encoding these two CXCR2 ligands

### Müller cells secrete IL-8 in response to stimulation with TNFα and IL-1β

Given our previous findings that hMC expression of CXCL8 and CXCL1 was upregulated by hMC stimulation with 1 ng/mL IL-1β and TNFɑ, and since hMC are known sources of VEGF, we assessed the protein levels of IL-8 (associated with DR in humans), CXCL1 (associated with OIR in mice), and VEGF in hMC-CM compared to the control unconditioned endothelial cell basal media (EBM).

**Fig. 1:**
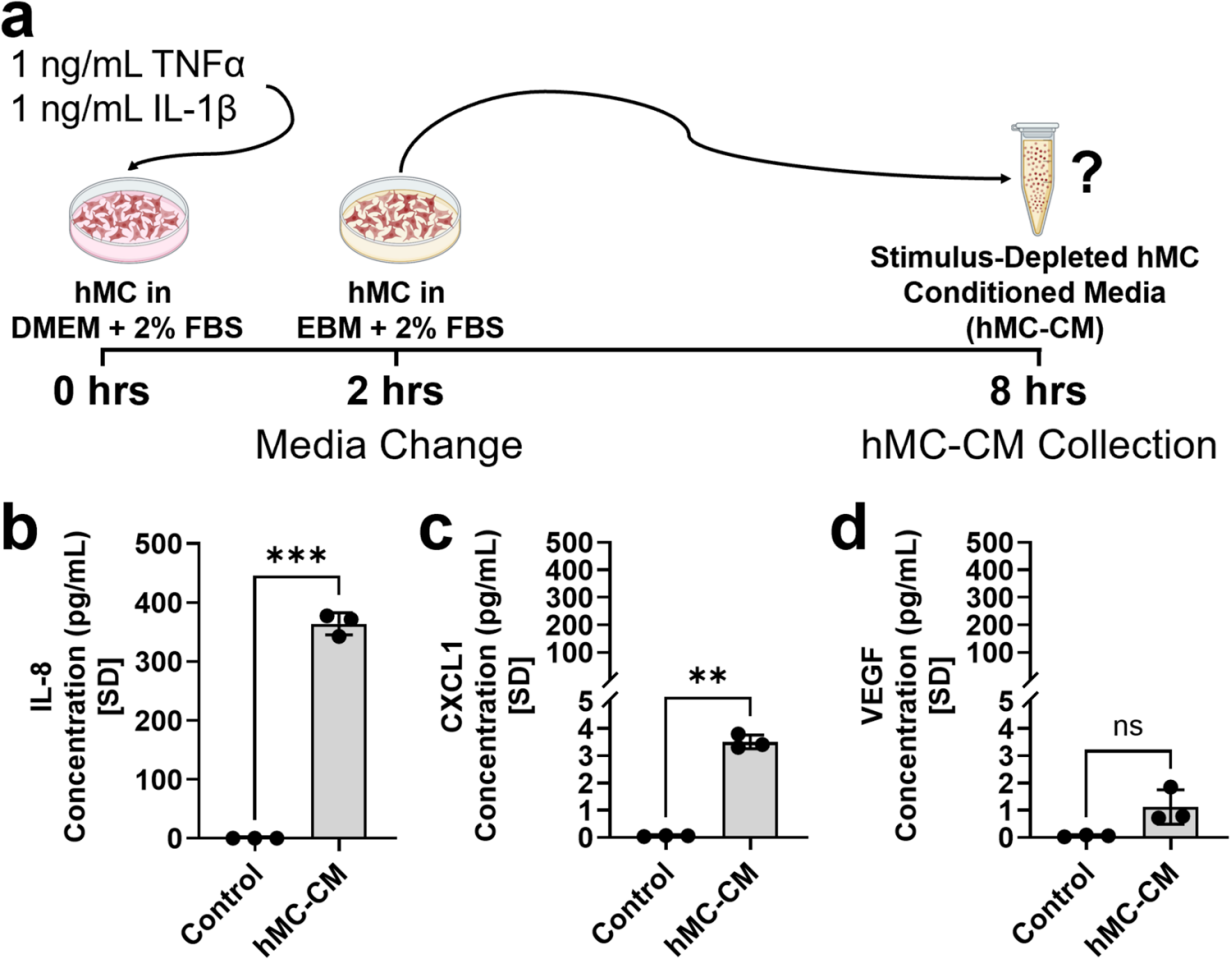
IL-8 and CXCL1, but not VEGF, are significantly upregulated in conditioned media from IL-1β and TNFα-stimulated hMC (hMC-CM). **a** Schematic displaying the generation of the stimulus-depleted hMC-CM after hMC are stimulated with 1 ng/mL IL-1β and TNFα for 2 h and the media changed to EBM. **b**–**d** Protein levels of **b** IL-8, **c** CXCL1, and **d** VEGF in unconditioned EBM (control) vs. hMC-CM. ELISA was conducted in three biological and technical replicates per group. Data were normally distributed, and two-tailed unpaired Welch’s t-tests were used. The threshold used for statistical significance was α = 0.05. Not significant, ns: *p* > 0.05, **: *p* ≤ 0.01, ***: *p* ≤ 0.001

To generate the hMC-conditioned media (hMC-CM), briefly, after hMC cultures were stimulated with 1 ng/mL TNFα and IL-1β for 2 h, the direct stimulus was depleted and the cells rinsed and cultured in EBM supplemented with 2% FBS for EBM-conditioning (Fig. 1a). This EBM, conditioned by secretions from hMC (hMC-CM; experimental), and the unconditioned EBM (control) were subjected to ELISA.

IL-8 protein levels were significantly elevated in hMC-CM but undetected in the control (*hMC-CM IL-8: 364.0* ± *18.67 pg/mL vs. Control IL-8: 0.032 pg/mL [extrapolated below the detection limit of the ELISA kit]; p* = *0.0009*; Fig. 1b). Although the protein concentration of CXCL1 was relatively low in both, it was sevenfold greater in hMC-CM than in unconditioned EBM (*hMC-CM CXCL1: 3.503* ± *0.2556 pg/mL vs. Control CXCL1: 0.05215* ± *0.02191 pg/mL; p* = *0.0017*; Fig. 1c). Finally, VEGF protein was below detection limits in both conditioned and unconditioned media (Fig. 1d).

**Fig. 2:**
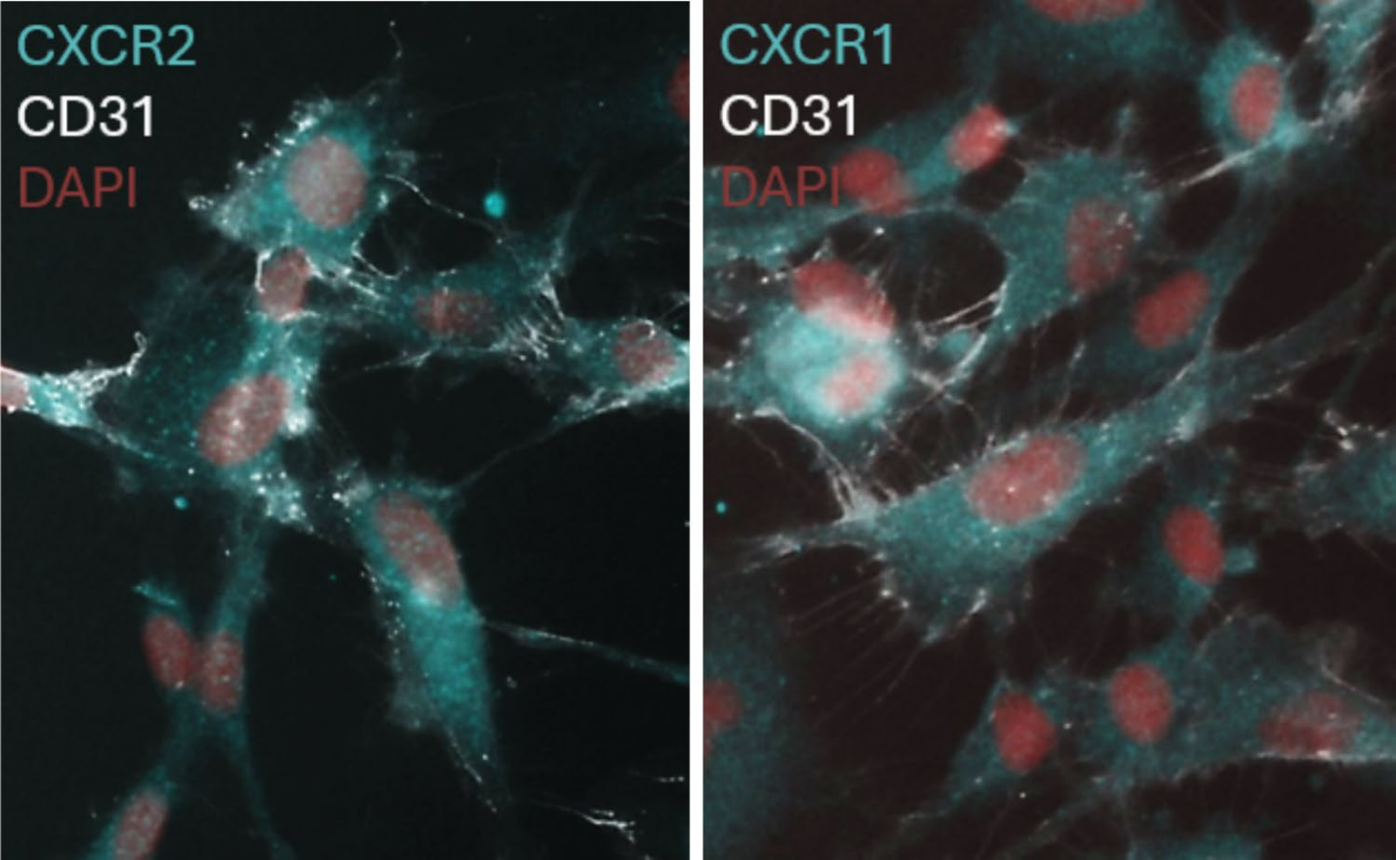
Human retinal microvascular endothelial cells (hRMEC) express CXCR1 and CXCR2. hRMEC cultures co-labeled with the anti-CD31 endothelial cell marker (gray), DAPI nuclear stain (red), and either anti-CXCR2 or anti-CXCR1 antibodies (cyan)

**Fig. 3:**
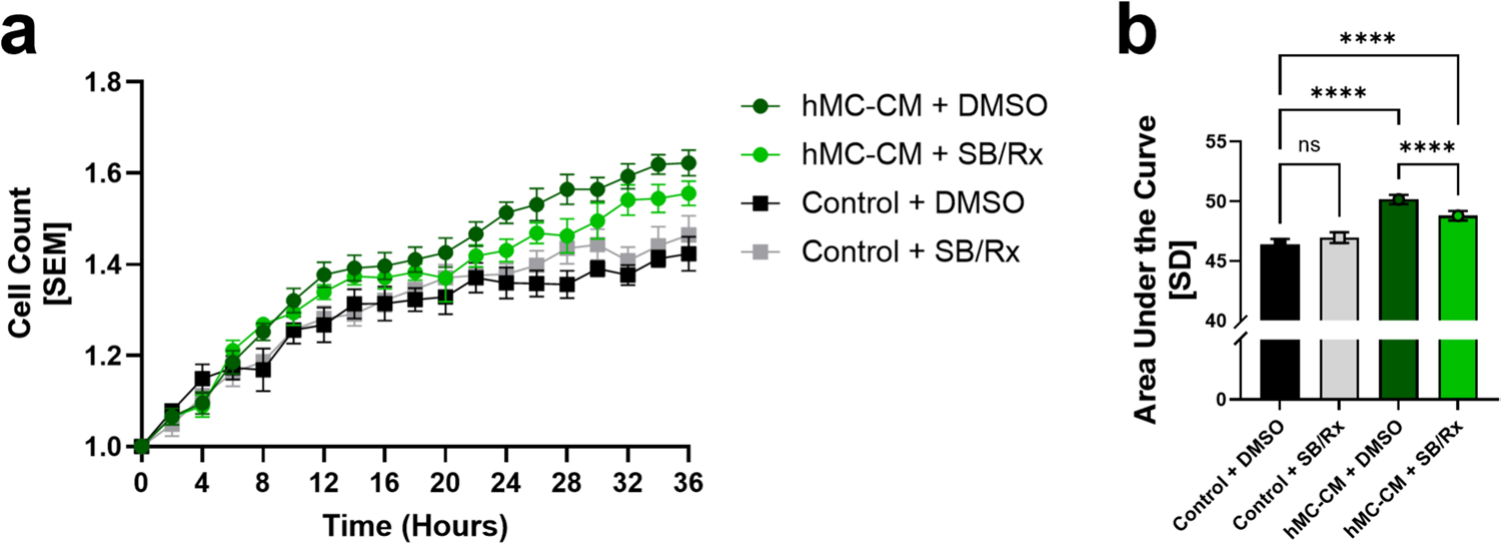
Conditioned media from IL-1β and TNFα-stimulated hMC (hMC-CM) enhances proliferation in hRMEC; CXCR1/2 inhibition decreases this effect. **a** Timelapse quantification of hRMEC proliferation following stimulation with hMC-CM vs. unconditioned EBM in the presence/absence of a combination of CXCR1/2 inhibitors (SB 50nM + Rx 75nM or “SB/Rx”). The cell count in each group was normalized to that group’s cell count after the addition of the unconditioned or conditioned media (n = 6 wells per group). **b** Area under the curve analysis of the proliferation time course. Data were normally distributed, and an ordinary One-Way ANOVA with Šídák multiple comparisons correction was used. The threshold used for statistical significance was α = 0.05. Not significant, ns: *p* > 0.05, ****: *p* ≤ 0.0001

Next, because CXCR2 ligands are known to be pro-angiogenic, we investigated the effect of the hMC-CM on endothelial cell angiogenic behaviors in vitro. hRMEC, whose expression of CXCR1 and CXCR2 was confirmed via immunostaining (Fig. 2), were treated with hMC-CM or control unconditioned EBM. Treatment with hMC-CM significantly induced hRMEC proliferation (area under the curve (AUC): *hMC-CM* + *DMSO: 50.16* ± *0.3909 vs. unconditioned EBM* + *DMSO: 46.40* ± *0.4479; p* < *0.0001*; Fig. 3a, b). Incubation with a cocktail of CXCR1/2 inhibitors—50 nM SB225002 (SB, a selective CXCR2 antagonist) in combination with 75 nM Reparixin (Rx, a dual CXCR1 and CXCR2 inhibitor)—significantly decreased the angiogenic effects of hMC-CM on hRMEC proliferation (AUC: *hMC-CM* + *DMSO: 50.16* ± *0.3909 vs. hMC-CM* + *SB/Rx: 48.80* ± *0.4011; p* < *0.0001*; Fig. 3a, b) relative to their vehicle (0.000381% DMSO). The presence of SB and Rx did not significantly alter the proliferation of hRMEC incubated in EBM alone.

**Fig. 4:**
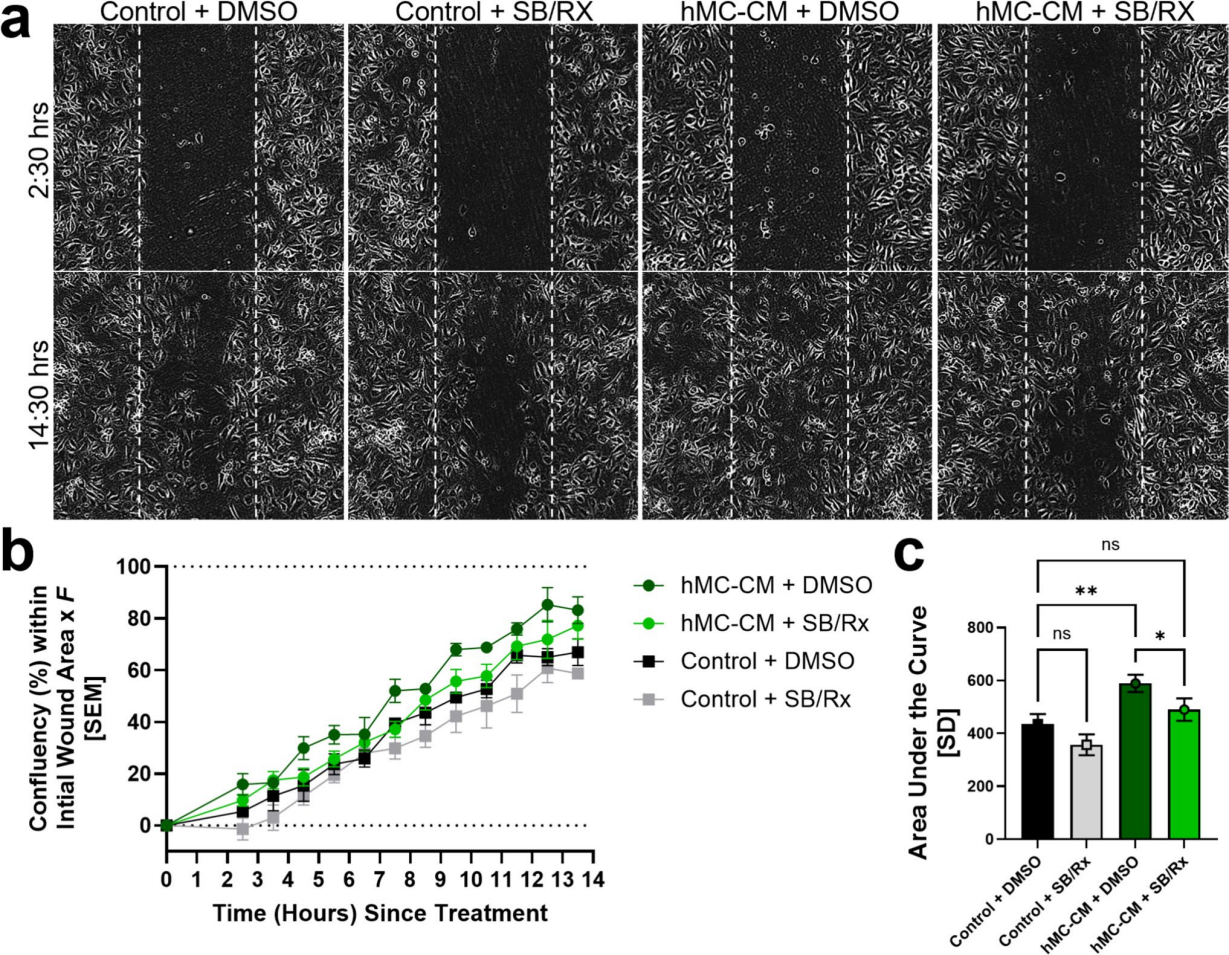
Conditioned media from IL-1β and TNFɑ-stimulated hMC (hMC-CM) enhances hRMEC migration; this effect is decreased by CXCR1/2 inhibition. **a** Representative images of wells, showing the initial hRMEC monolayer scratch/wound 2.5 h after treatment and the confluency of cells within the wound 13.5 h after treatment. **b** Confluency time course within the initial hRMEC monolayer scratch/wound. The initial wound area of each well was defined as 0% directly after the addition of treatments and 100% once the wound area was fully confluent. To account for discrepancies between machine-calculated and observed confluency, all data were multiplied by the constant wound factor *F* such that a fully-confluent well corresponds to a confluency of 100%. Treatment groups included hMC-CM vs. unconditioned EBM control, each in the presence of a combination of CXCR1/2 inhibitors (SB 50 nM and Rx 75 nM “SB/Rx”) or their vehicle, 0.000381% DMSO (n = 3–4 wells per group). **c** Area under the curve analysis of the migration time course for each group. Data were normally distributed, and an ordinary One-Way ANOVA with Šídák multiple comparisons correction was used. The threshold used for statistical significance was α = 0.05. Not significant, ns: *p* > 0.05, *: *p* ≤ 0.05, **: *p* ≤ 0.01

Since angiogenesis involves both endothelial cell proliferation and migration, we also performed hRMEC scratch/wound healing assays after treatment with hMC-CM vs. the unconditioned EBM control. In addition, we compared the ability of CXCR1/2 inhibitors vs. their vehicle (0.000381% DMSO) to block these effects (Fig. 4a). Wound closure, represented by higher hRMEC confluency within the region of the initial scratch/wound, was also significantly faster (higher confluency) in hRMEC treated with the hMC-CM vs. the unconditioned EBM (AUC: *hMC-CM* + *DMSO: 588.9* ± *32.60 vs. unconditioned EBM* + *DMSO: 435.3* ± *37.66; p* = *0.0015*; Fig. 4b, c). This effect was more pronounced than the effect on hRMEC proliferation. Furthermore, incubation with SB/Rx significantly reduced the effect of hMC-CM on hRMEC migration, reducing hRMEC confluency within the wound (AUC: *hMC-CM* + *DMSO: 588.9* ± *32.60 vs. hMC-CM* + *SB/Rx: 490.1* ± *42.34; p* = *0.0286*; Fig. 4b, c). The addition of SB/Rx did not have a significant effect on the migration of hRMEC treated with unconditioned EBM.

The findings that hRMEC proliferation and migration were stimulated by hMC-CM suggest that hMC are a paracrine source of angiogenic signals. In addition, the finding that CXCR1/2 inhibitors mitigated the hMC-CM effects on hRMEC proliferation and migration suggests that at least part of the hMC-CM’s angiogenic effect was due to the presence of CXCR1/2 ligands. Since IL-8 was highly upregulated in the hMC-CM while VEGF was undetectable, we sought to isolate and evaluate the potential pro-angiogenic properties of this high affinity CXCR2 ligand.

### IL-8 stimulates hRMEC proliferation and migration

**Fig. 5:**
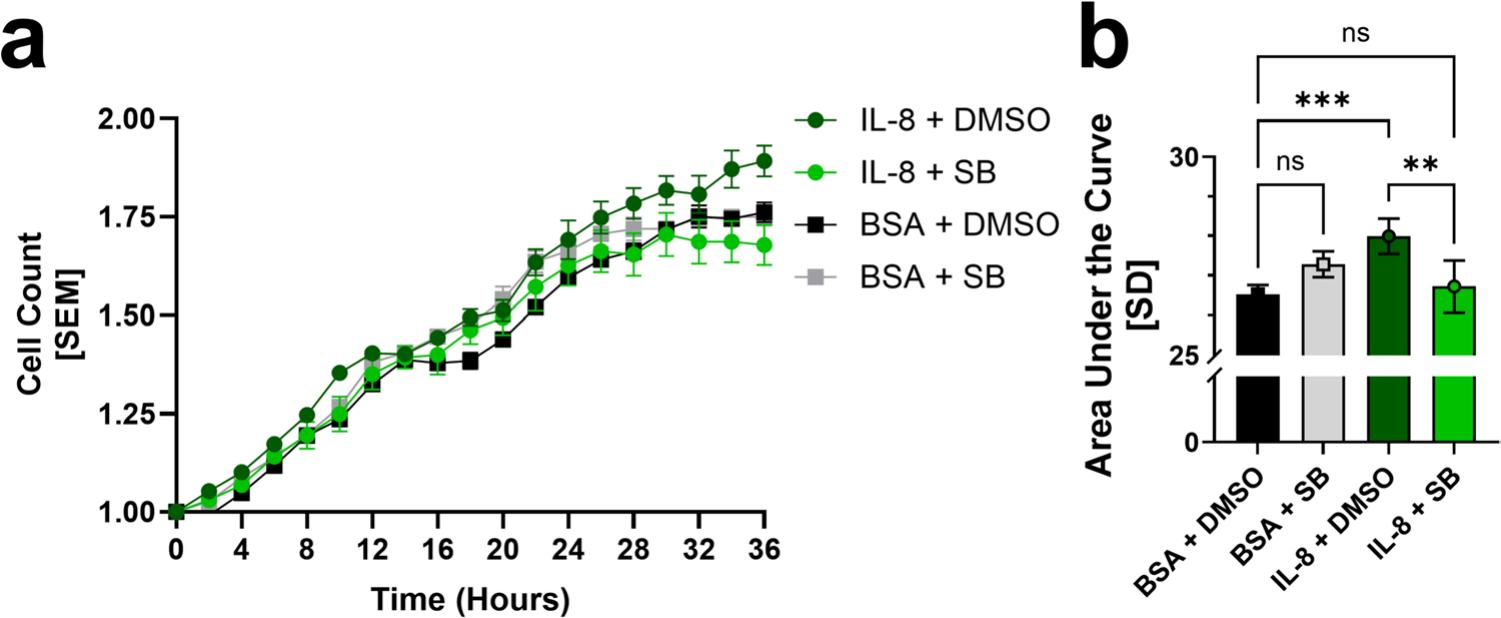
Direct stimulation of hRMEC with IL-8 enhances their proliferation; the CXCR2 inhibitor, SB225002, inhibits this induction. **a** Timelapse quantification of hRMEC proliferation following stimulation with 25 ng/mL IL-8 vs. BSA control in the presence and absence of the CXCR2-specific inhibitor SB225002 at 50 nM (n = 5 wells per group). The cell count in each group is normalized to that group’s cell count after addition of the 25 ng/mL of IL-8 or BSA stimulus. **b** Area under the curve analysis of the proliferation time course of each group. Data were normally distributed, and an ordinary One-Way ANOVA with Šídák multiple comparisons correction was used. The threshold used for statistical significance was α = 0.05. Not significant, ns: *p* > 0.05, **: *p* ≤ 0.01, ***: *p* ≤ 0.001

IL-8 has been shown to directly stimulate proliferation and migration in endothelial cells from several organ systems, but its effects on microvascular cells from human retinas have not been reported. We thus compared the ability of human recombinant IL-8 (25 ng/mL) vs. its vehicle (0.1% BSA) to induce proliferation and migration of hRMEC in vitro. In addition, we tested whether CXCR2 inhibition could reduce these effects. Stimulation with IL-8 significantly increased hRMEC proliferation compared to its BSA control (AUC: *IL-8* + *DMSO: 27.99* ± *0.4477 vs. BSA* + *DMSO: 26.52* ± *0.2337; p* = *0.0003*; Fig. 5a, b). The addition of the CXCR2 inhibitor SB225002 significantly decreased the proliferation induced by IL-8 (AUC: *IL-8* + *DMSO: 27.99* ± *0.4477 vs. IL-8* + *SB: 26.72* ± *0.6523; p* = *0.0014*; Fig. 5a, b) and did not significantly alter the level of proliferation associated with BSA control stimulation.

**Fig. 6:**
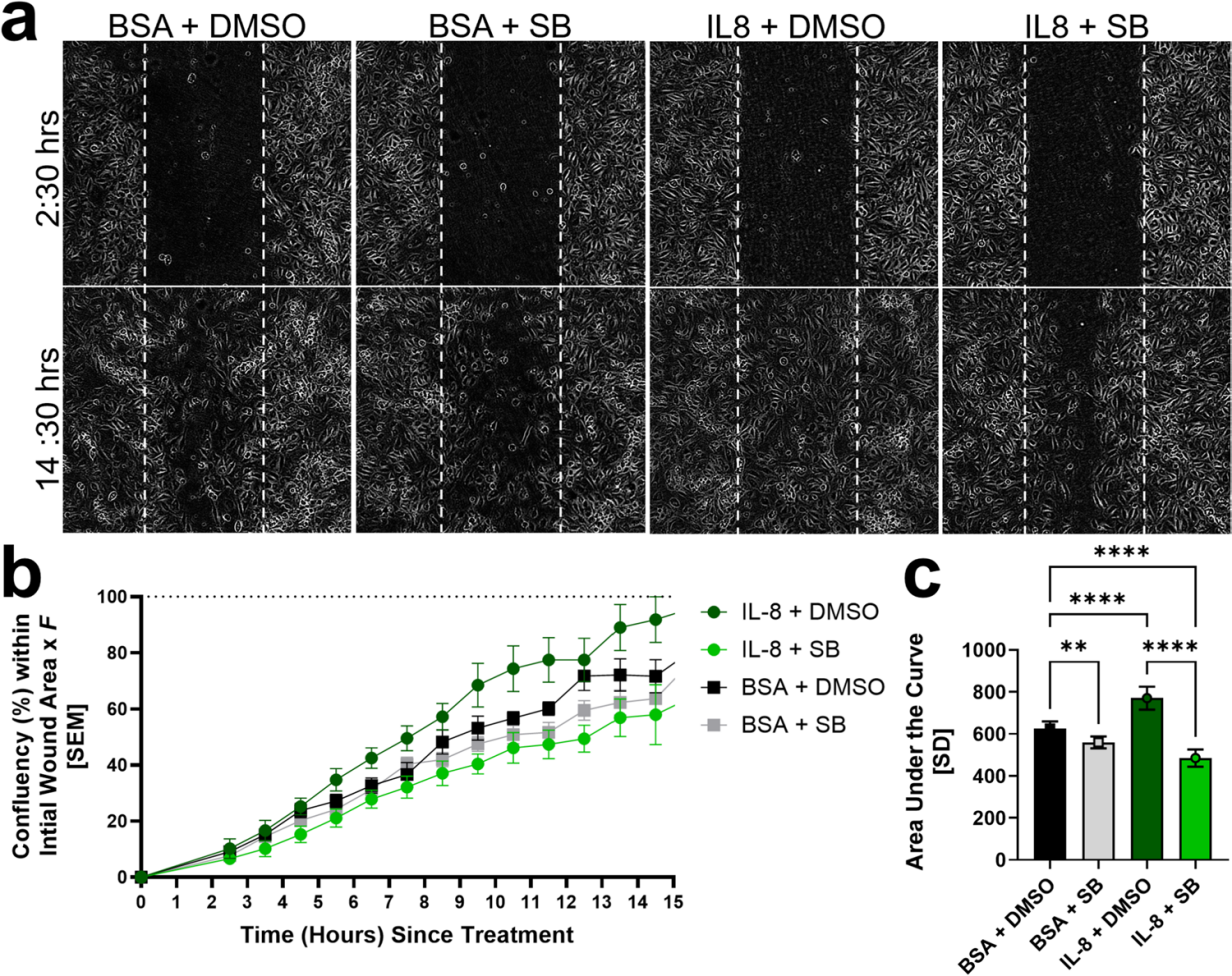
Direct stimulation of hRMEC with IL-8 enhances hRMEC migration; co-treatment with SB225002 completely blocks this effect. **a** Representative images of wells, showing the initial hRMEC monolayer scratch/wound 2.5 h after treatment and the confluency of cells within the wound 14.5 h after treatment. **b** Confluency time course within the initial hRMEC monolayer scratch/wound. The initial wound area of each well was defined as 0% directly after the addition of treatments and 100% once the wound area was fully confluent. To account for discrepancies between machine-calculated and observed confluency, all data were multiplied by the constant wound factor *F* such that a fully-confluent well corresponds to a confluency of 100%. Treatment groups included 25 ng/mL IL-8 in BSA vs. its BSA control, each in the presence of 50 nM SB225002 in DMSO or its vehicle, *0.0000587%* DMSO (n = 9–11 wells per group). **c** Area under the curve analysis of the migration time course of each group. Data were normally distributed, and an ordinary One-Way ANOVA with Šídák multiple comparisons correction was used. The threshold used for statistical significance was α = 0.05. Not significant, ns: *p* > 0.05, **: *p* ≤ 0.01, ****: *p* ≤ 0.0001

As observed with hMC-CM, in which IL-8 levels were significantly upregulated, direct stimulation with IL-8 also significantly enhanced hRMEC migration, increasing confluency within the wound (AUC: *IL-8* + *DMSO: 770.2* ± *54.49 vs. BSA* + *DMSO: 625.3* ± *34.13*; *p* < 0.0001; Fig. 6a–c). The addition of SB significantly attenuated the pro-migratory effect of IL-8 stimulation (AUC: *IL-8* + *DMSO: 770.2* ± *54.49 vs. IL-8* + *SB: 485.3* ± *41.34*; *p* < 0.0001; Fig. 6c).

**Fig. 7:**
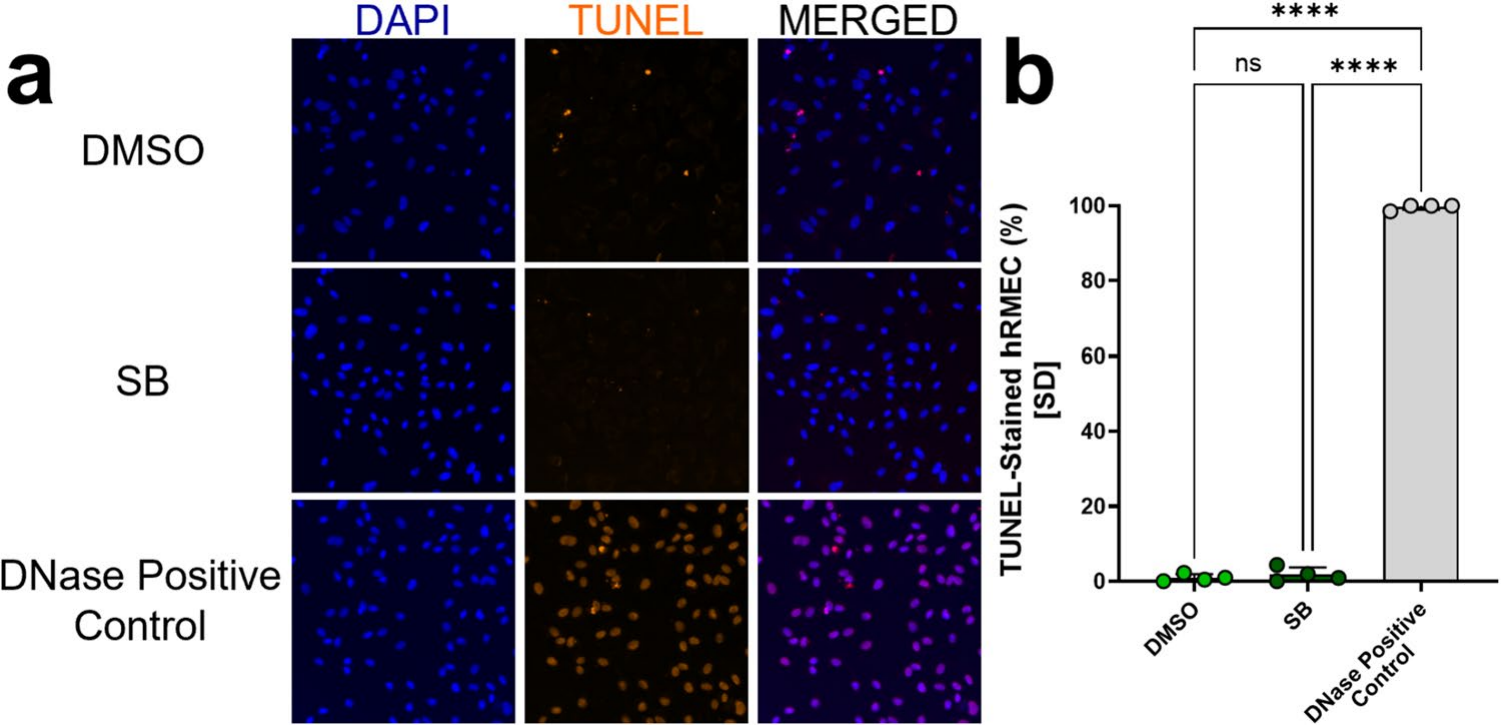
Incubation with SB225002 or its vehicle, DMSO, does not induce hRMEC death. **a** hRMEC stained with DAPI and the TUNEL reaction reagent (see methods for details) show healthy cells in the presence of SB and its control. **b** Analysis of the percentage of TUNEL-positive hRMEC shows that the TUNEL stain, which worked effectively in the positive control DNase group, did not colocalize with the nuclei of hRMEC treated with 50nM SB (in 0.0000587% DMSO) or 0.0000587% DMSO alone (n = 4 images from 2 wells for each group). Data were normally distributed, and an ordinary One-Way ANOVA with Šídák multiple comparisons correction was used. The threshold used for statistical significance was α = 0.05. Not significant, ns: *p* > 0.05, ****: *p* ≤ 0.0001

Because SB demonstrated a significant inhibitory effect even in unstimulated media and because proliferation and migration could have been affected by cell death, we examined the possibility of SB-induced hRMEC death. Neither 50 nM SB, which is reconstituted in DMSO, nor its vehicle (0.0000587% DMSO) induced significant cell death in the hRMEC (%TUNEL^+^ cells: *DMSO: 0.91* ± *0.98% vs. DNase positive control: 99.63* ± *0.75%, p* < *0.0001; SB: 1.79* ± *1.88% vs. DNase: 99.63* ± *0.75%, p* < *0.0001*; Fig. 7a, b).

### CXCR2 activation modulates pathological neovascularization

**Fig. 8:**
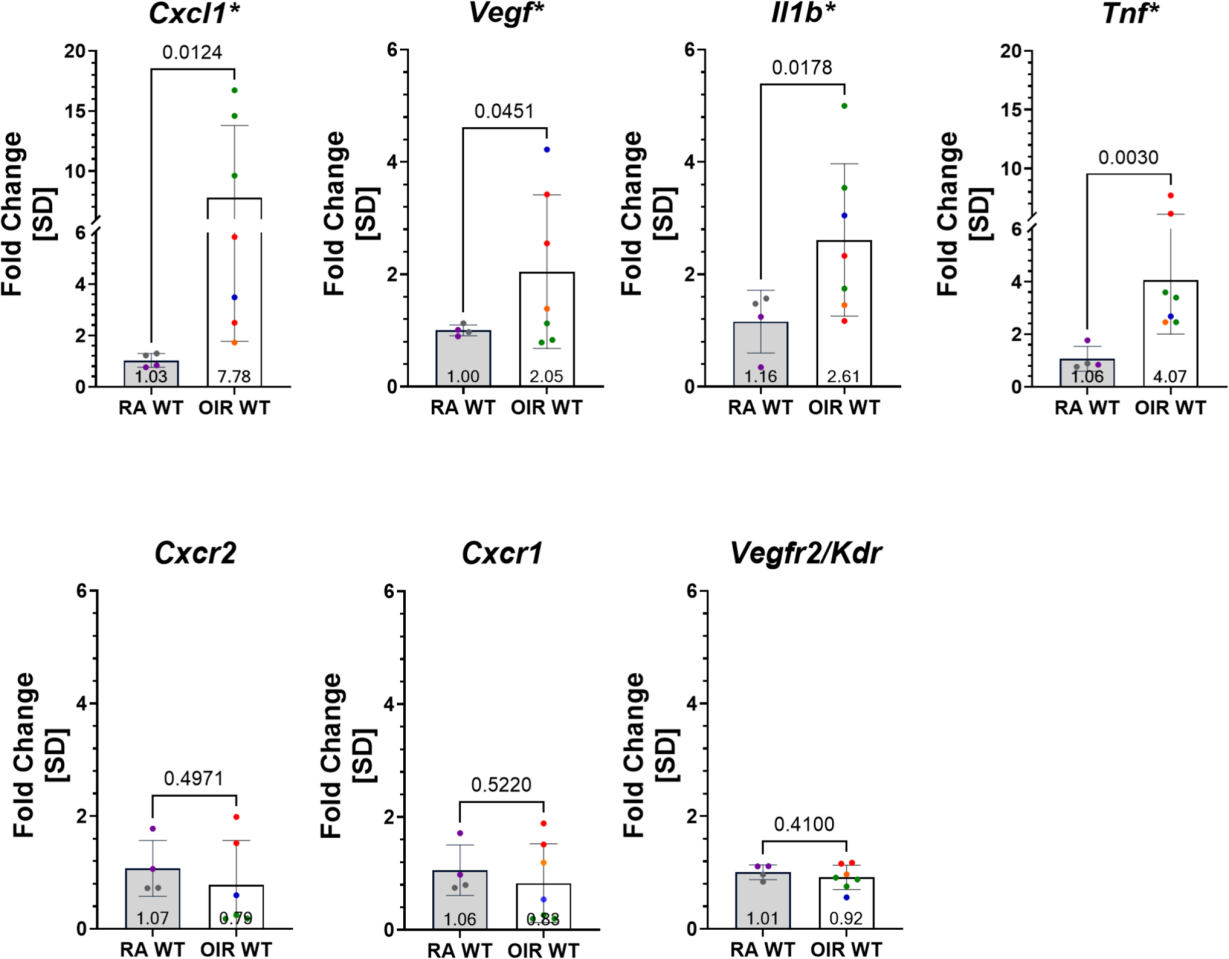
Retinal expression of *Cxcl1*, *Vegf*, *Il1b*, and *Tnf* was significantly upregulated in mice subjected to OIR as compared to mice in normoxia. qRT-PCR analysis of relative expression levels of key inflammatory and angiogenic cytokines and receptors in OIR vs. normoxia/room air (RA). The fold change of each gene was normalized to the RA samples of each gene, with different colors representing data from distinct litters. Seven transcripts of interest were compared. There was a significant upregulation for *Cxcl1*, *Vegf*, *Il1b*, and *Tnf* in OIR mice. If the fold change data for a given gene were normally distributed, a Welch’s two-tailed unpaired t-test was used, and if the data were not normally distributed, a two-tailed, unpaired Mann Whitney test was used. The threshold for statistical significance was α = 0.05

In mice, the IL-8 homologue, CXCL1, has been shown to be upregulated both after intravitreal injection of IL-1β [28] and in OIR, where its expression maps predominantly to Müller cells [19]. We compared the expression of *Cxcl1*, *Cxcr1*,* Cxcr2*, *Vegf*, *Vegfr2/Kdr*, *Il1b*, and *Tnf* transcripts at ~ p17 between mice reared in room air and those subjected to OIR conditions between postnatal days 7–12. Using quantitative real-time PCR (qRT-PCR), we confirmed significant upregulation of *Cxcl1* (Fold change (FC) *RA WT: 1.0268* ± *0.2685 vs. OIR WT: 7.7763* ± *6.0064, p* = *0.0124)*, *Vegf* (FC *RA WT: 1.0034* ± *0.0963 vs. OIR WT: 2.0479* ± *1.3677, p* = *0.0451*), *Il1b* (FC *RA WT: 1.1596* ± *0.5592 vs. OIR WT: 2.6133* ± *1.3552, p* = *0.0178*), and *Tnf* (FC *RA WT: 1.0638* ± *0.4738 vs. OIR WT: 4.0653* ± *2.0580, p* = *0.0030*) in mice exposed to hyperoxia (in OIR) versus room air (RA) (Fig. 8).

**Fig. 9:**
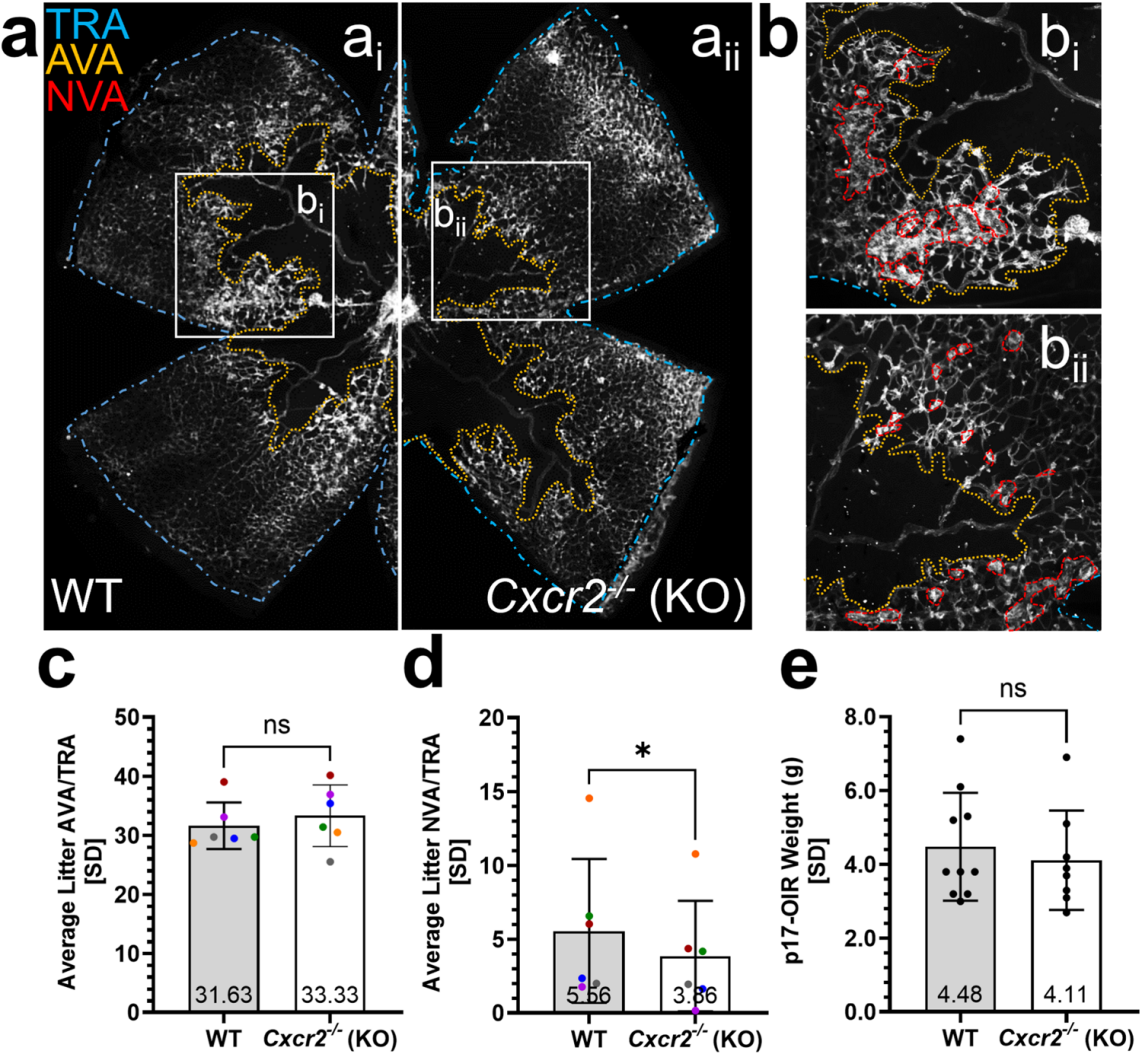
Reduced neovascularization in *Cxcr2*^*-/-*^ compared to wild-type OIR mice. **a** Example of wild-type (a_i_) and *Cxcr2*^*-/-*^ (a_ii_) retinal flat mounts in one of the six OIR sessions at p17-OIR. Small white boxes indicate selected regions for which neovascularization (NV) is outlined in larger insets (b_i_, b_ii_). Total retinal area (TRA), avascular area (AVA), and neovascular area (NVA) are indicated in turquoise, orange, and red, respectively; these were traced by a masked observer. **c** The avascular area was not significantly different between the wild-type and *Cxcr2*^*-/-*^ retinas. Data were not normally distributed, and a Wilcoxon two-tailed paired t-test was used. **d** *Cxcr2*^*-/-*^ mice exhibited a 30.6% reduction in NV compared to WT. Data were normally distributed, and a paired two-tailed t-test was used. **e** There was no statistically significant difference between the body weights of p17-OIR WT and *Cxcr2*^*-/-*^ mice. Data were normally distributed, and a Welch’s two-tailed unpaired t-test was used. For both the AVA/TRA and NVA/TRA analyses, data were collected from both retinas of 10 WT and 8 *Cxcr2*^*-/-*^ littermate mice from 6 litters. WT and *Cxcr2*^*-/-*^ data were averaged separately for each litter, with different colors representing experimental and control group averages from distinct litters. The threshold for statistical significance was α = 0.05. ns: *p* > 0.05, *: *p* ≤ 0.05

Given our suspicion that CXCL1 upregulation may link inflammatory responses, such as elevated IL1β and TNFα, to the induction of retinal angiogenesis, we then sought to evaluate the effects of inhibiting CXCR2 signals on pre-retinal NV in OIR. In this model, the window of upregulation of CXCL1 is reported to coincide with retinal angiogenesis and to map to Müller cells [19]. Since CXCR2-mediated inflammatory responses (i.e., neutrophil chemotaxis and degranulation) could affect the OIR phenotype, we first explored the effects of genetic inactivation of CXCR2 on pre-retinal NV by inducing OIR in unperturbed *Cxcr2*^*-/-*^ vs. WT littermate mice. This avoided any manipulation (e.g., intravitreal injection) that could induce inflammation in the eye and confound our interpretation of OIR results. We compared the percentages of both avascular and neovascular retinal areas (AVA and NVA, respectively) relative to the total retinal area (TRA) of each flat mount between *Cxcr2*^*-/-*^ and WT littermate mice at p17-OIR (Fig. 9a, b). Although no significant difference was observed in AVA (Fig. 9c), the NVA was significantly smaller (30.6% reduction) in the *Cxcr2*^*-/-*^ compared to wild-type mice (p17-OIR *NVA/TRA Cxcr2*^*-/-*^*: 3.857%* ± *3.755 vs. WT: 5.561%* ± *4.881; p* = *0.0236*; Fig. 9d).

As is the case with premature babies with lower birthweight who are at risk for worse retinopathy of prematurity, lower body weight in mice has been associated with worse OIR at p17 [31]. In our experiments, the degree of NV observed was in line with published expectations for pups weighing 4-5g [32], and weights were not significantly different between KO and WT control littermates (p17-OIR *body weight: Cxcr2*^*-/-*^*: 4.11g* ± *1.3442 vs. WT: 4.48g* ± *1.4604; p* = *0.5870*; Fig. 9e).

We expected the neovascular area to be proportional to avascular area, but there was no difference in AVA at p17 despite the > 30% reduced NV. Because the avascular area at p12 (at the transition from hyperoxia to normoxia) is more likely to influence the NV at p17, we also compared AVA/TRA at p12. We did not detect a significant difference in AVA between *Cxcr2*^*-/-*^ and WT retinas at p12 (p12-OIR *AVA/TRA Cxcr2*^*-/-*^*: 35.66%* ± *2.005 vs. WT: 37.22%* ± *3.081; p* = *0.2032*, Paired Two-Tailed t-test), suggesting that CXCR2 inhibition is uncoupling the expected relationship between AVA and NVA.

**Fig. 10:**
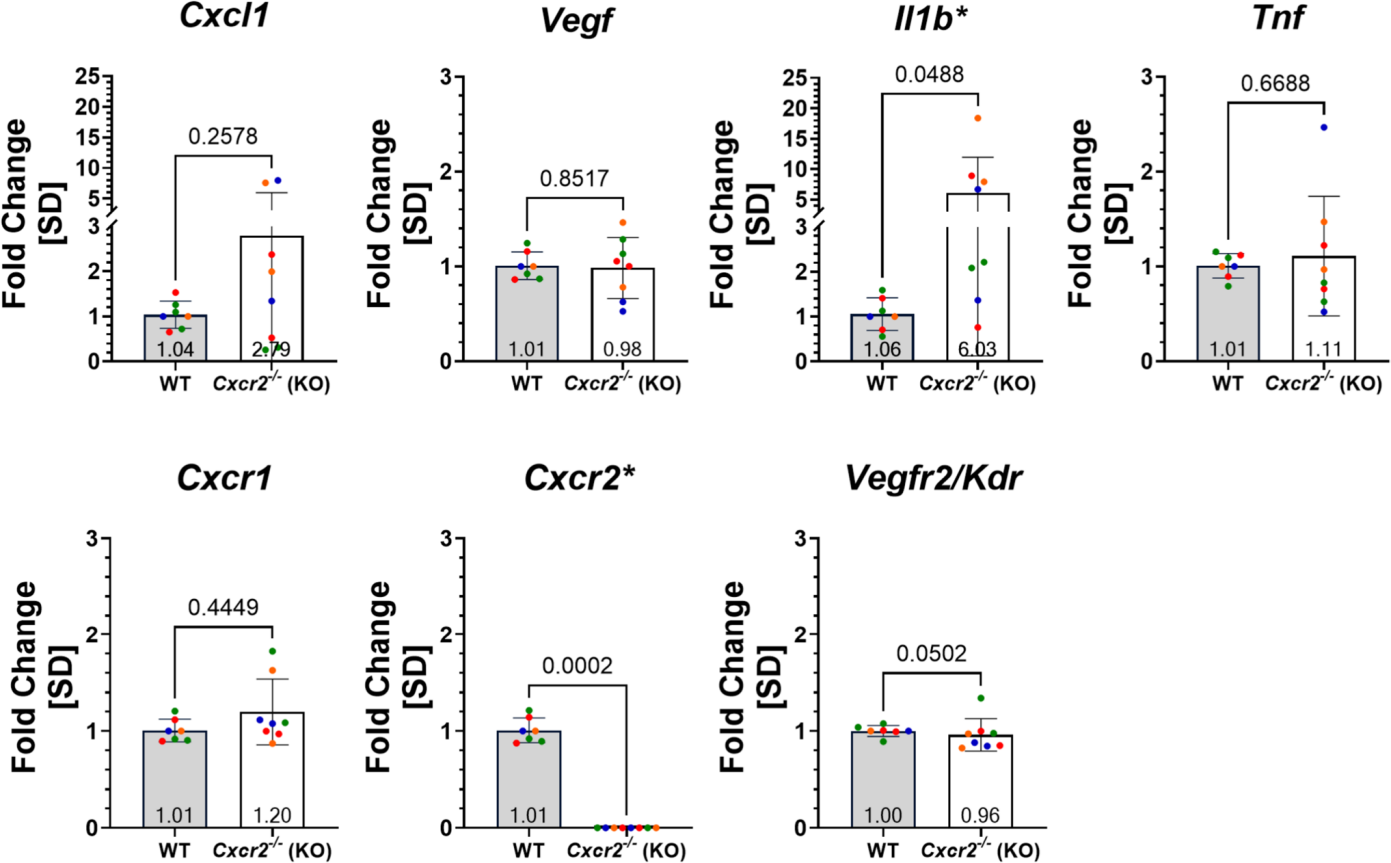
*Il1b* is upregulated in the retinas of *Cxcr2*^-/-^ mice at p17-OIR. qRT-PCR analysis of relative expression levels of key inflammatory and angiogenic cytokines and receptors was used to compare *Cxcr2*^*-/-*^ and WT responses in OIR at p17. The fold change of each gene was normalized to the WT of each litter, with different colors representing data from distinct OIR litters. Seven transcripts of interest were compared. There was a trend towards upregulation for *Cxcl1* and *Il1b*, which was only statistically significant for *Il1b*. In addition, PCR for *Cxcr2* confirmed the CXCR2 KO status. Finally, although *Cxcr2*^*-/-*^ mice showed decreased NV, there was no detectable change in their retinal levels of *Vegf* expression. A Welch’s two-tailed unpaired t-test was used when the fold change data for a given gene were normally distributed, and a two-tailed unpaired Mann Whitney test was used when the data were not normally distributed. The threshold for statistical significance was α = 0.05

To better understand whether this change in NV was related to changes in expression of VEGF or other inflammatory and angiogenic cytokines and their receptors of interest, we performed qRT-PCR on retinas from p17-OIR *Cxcr2*^*-/-*^ and WT littermate mice. As expected, *Cxcr2* was not expressed in *Cxcr2*^*-/-*^ mice (Fig. 10). In addition, only *Il1b* was significantly upregulated in *Cxcr2*^*-/-*^ mice (FC *Il1b* p17-OIR *WT: 1.0568* ± *0.3645 vs. Cxcr2*^*-/-*^*: 6.0300* ± *5.9054; p* = *0.0488)*. Finally, expression of *Vegf* and its receptor was similar between WT and *Cxcr2*^*-/-*^ mice, suggesting that the decreased NV in the *Cxcr2*^*-/-*^ mice was VEGF independent.

**Fig. 11:**
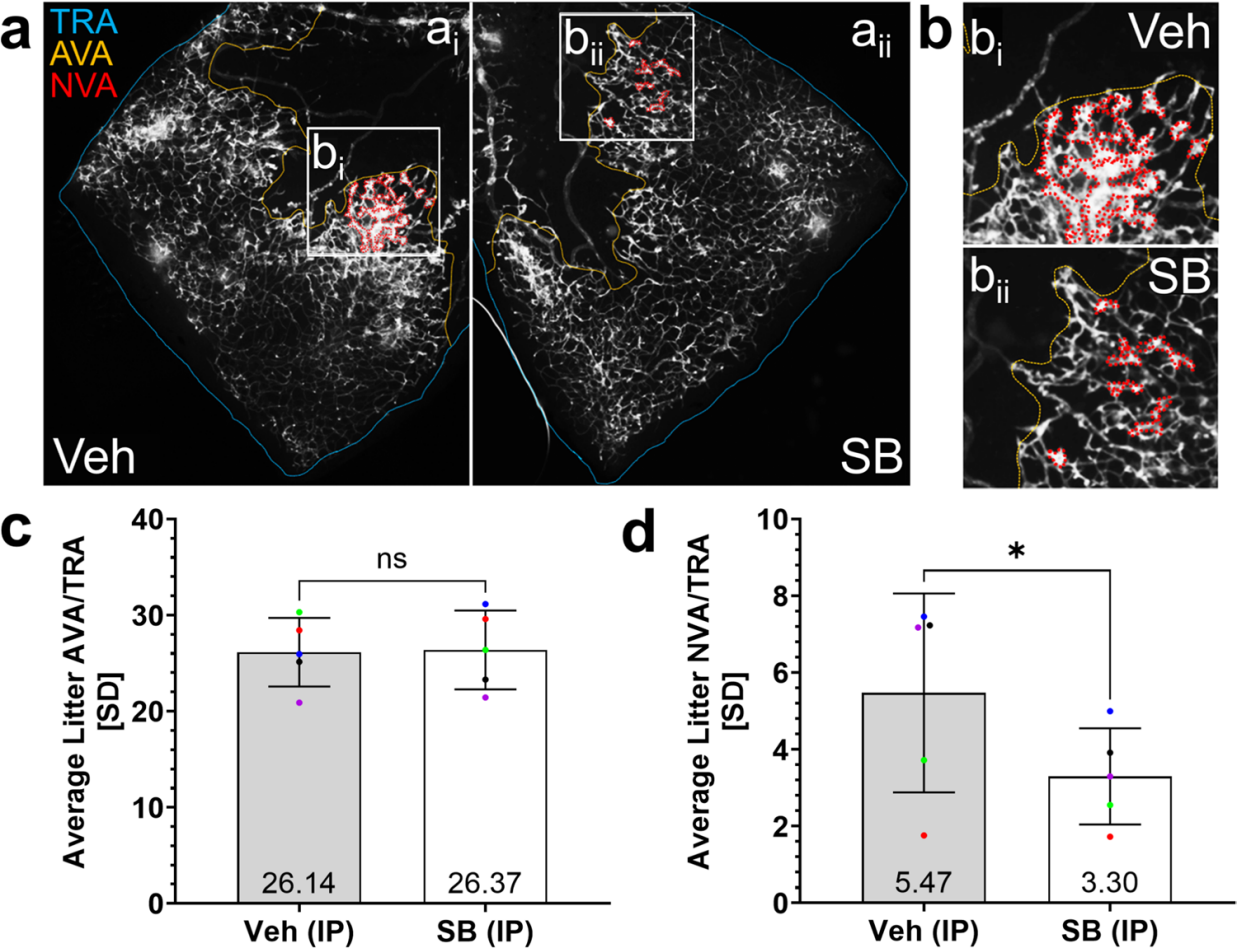
Reduced neovascularization in OIR in wild-type mice receiving systemic SB225002. **a** Representative flat mount images of retinas from WT mice administered daily IP injections of 0.008% DMSO vehicle (a_i_) or 4mg/kg SB (a_ii_). **b** Small white boxes indicate selected regions for which NV is outlined in larger insets (b_i_, b_ii_). TRA, AVA, and NVA are indicated in turquoise, orange, and red, respectively. **c** The avascular area was not significantly different between retinas of mice administered daily IP injections of SB at 4mg/kg or 0.1% DMSO. Data were normally distributed, and a paired two-tailed t-test was used. **d** Mice administered IP injections of SB exhibited a 39.7% reduction in NV compared to those given vehicle. Data were normally distributed, and a paired two-tailed t-test was used. For both the AVA/TRA and NVA/TRA analyses, data were collected from both retinas of 12 mice that received IP injections of 4 mg/kg SB and 11 mice that received IP injections of 0.1% DMSO from 5 litters. AVA and NVA data for mice administered vehicle and SB injections were averaged separately for each litter, with different colors representing experimental and control group averages from distinct litters. The threshold for statistical significance was α = 0.05. ns: *p* > 0.05, *: *p* ≤ 0.05

After establishing that genetic deletion of CXCR2 decreased pre-retinal NV, we wanted to test the effects of pharmacologic inhibition of CXCR2, as this would better mimic therapies for humans. To do so, we administered daily intraperitoneal injections of the CXCR2 inhibitor SB225002 at 4 mg/kg in wild-type mice between p12-p17 of OIR, the post-exposure period between return to room air and euthanasia. As with the KO vs. WT experiments, retinal flat mounts were traced by a masked observer to outline AVA, TRA, and NVA (Fig. 11a, b). As seen in the KO mice, while there was no difference in the relative avascular area between mice treated with 4mg/kg SB225002 or its vehicle, 0.008% DMSO (Fig. 11c), we detected significantly decreased (~ 40% lower) NV in the mice receiving daily intraperitoneal injections of SB225002 vs. vehicle (p17-OIR *NVA/TRA: SB (IP): 3.296%* ± *1.253 vs. Vehicle (IP): 5.468%* ± *2.589; p* = *0.0367*; Fig. 11d). As with the KO, this reduced NV occurred irrespective of mouse body weight (p17-OIR *body weight: SB (IP): 4.31* ± *0.7279g vs. Vehicle (IP): 4.10* ± *0.8042g; p* = *0.6032, data not shown*)*.*

**Fig. 12:**
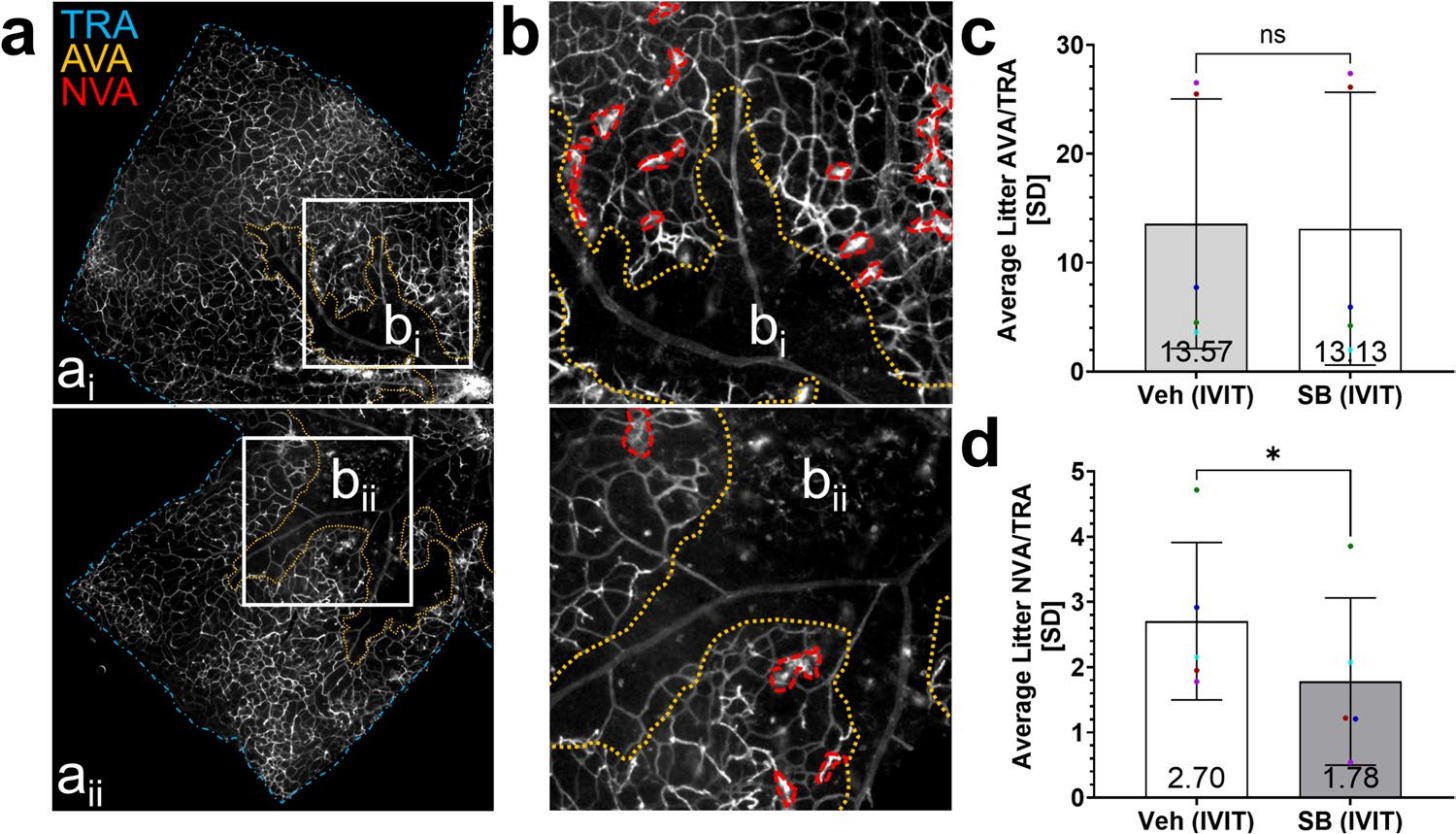
Pre-retinal neovascularization is significantly reduced in OIR mice after intravitreal injections of the CXCR2 inhibitor SB225002. **a** Representative flat mount images of retinas from eyes of mice administered contralateral IVIT injections of 0.0017605% DMSO vehicle (a_i_) and 1.5µM SB (a_ii_). **b** Small white boxes indicate selected regions for which NV is outlined in larger insets (**b**_i_, b_ii_). **c** The avascular area was not significantly different between retinas of mice administered IVIT injections of 1.5µM SB or 0.0017605% DMSO. Data were normally distributed, and a paired two-tailed t-test was used. **d** Mice administered IVIT injections of 1.5µM SB225002 exhibited a 34.2% reduction in neovascular area compared to those given 0.0017605% DMSO vehicle. Data were normally distributed, and a paired two-tailed t-test was used. For both the AVA/TRA and NVA/TRA analyses, data were collected from retinas of 10 mice that received contralateral IVIT injections of 1.5µM SB and 0.0017605% DMSO from 5 litters. AVA and NVA data for mice administered vehicle and SB injections were averaged separately for each litter, with different colors representing experimental and control group averages from distinct litters. The threshold for statistical significance was α = 0.05. ns: *p* > 0.05, *: *p* ≤ 0.05

We next explored the ability of intravitreal SB injections to reduce pre-retinal NV in OIR. This local administration was expected to increase the concentration of available drug and minimize potential off-target effects. Wild-type mice were given intravitreal injections of 1.5µM SB in one eye and 0.0017605% DMSO vehicle in the other at p12-OIR, the time of removal from the hypoxic OIR chamber. This contralateral eye treatment allowed us to also control for any innate differences between animals, including variations in body weight. Eyes were harvested at p17-OIR, and the retinas were dissected, stained, flat mounted, imaged, and traced by a masked observer (Fig. 12a, b). As seen in our prior experiments with genetic or pharmacologic CXCR2 inhibition, the AVA was not significantly different between the two groups (Fig. 12c), while the NVA was significantly decreased (34%) in retinas from eyes that received intravitreal (IVIT) injections of 1.5µM SB225002, compared to those that received 0.0017605% DMSO control (*NVA/TRA: SB (IVIT): 1.780%* ± *1.282 vs. Vehicle (IVIT): 2.704%* ± *1.206; p* = *0.0271*; Fig. 12d). The NV observed after ocular puncture was reduced as expected from reports by Penn et al. [33].

## Discussion

Diabetic retinopathy (DR) is characterized by chronic inflammation and pre-retinal NV leading to vision loss. The inflammatory cytokines IL-1β, TNFα, and IL-8 have been consistently identified in ocular samples of patients with DR. They accumulate in fluids and tissues of eyes with DR along with VEGF—the target of currently approved DR drugs—and, like VEGF, may also play important roles in DR pathophysiology. However, how the interplay of these signals affects NV is not well understood.

We previously demonstrated direct and paracrine upregulation of IL-8 and other CXCR2 ligands in hRMEC and hMC following IL-1β and TNFα stimulation [28]. In addition, our group has also shown upregulation of CXCR2 ligands in microglia, Müller cells, and astrocytes following stimulation with palmitic acid [27]. Palmitic acid is a detrimental free fatty acid elevated in hyperlipidemia, a common diabetes comorbidity associated with worse DR outcomes. Furthermore, others have demonstrated retinal cell upregulation of CXCR2 ligands following stimulation with TNFα and with other DR-associated signaling molecules, including VCAM [34–36].

IL-8 is a potent neutrophil chemoattractant. Neutrophil degranulation, which is also enhanced by TNFα and IL-8, promotes matrix metalloproteinase (MMP) release, which can, in turn, activate pro-forms (e.g., pro-IL-1β to IL-1β), modify chemokines (amino terminal cleavage and activation of IL-8), and cleave extracellular matrix tethers of key pathogenic signaling intermediates (e.g., VEGF untethering), thereby leading to disease progression [37–40]. MMP-mediated activation of IL-1β could lead to amplification and propagation of inflammatory responses in the retina, while VEGF untethering may make it more accessible to induce NV after intravitreal accumulation. Thus, IL-8, which itself can possess both pro-inflammatory and pro-angiogenic activities, may promote angiogenesis both directly and indirectly by recruiting immune cells and promoting the activation and release of pathogenic molecules that contribute to DR progression. In turn, TNFα, whose serum levels correlate with DR [41], has been shown to enhance IL-8 expression in glia, endothelial cells, and neutrophils [36, 42].

CXCR2 activation by IL-8 has been extensively investigated in cancer and other inflammatory, angiogenic, and fibrotic diseases [16, 17, 20, 43–55]. Although prior studies have implicated expression of CXCR2 ligands in DME, hypertensive retinopathy, and choroidal NV [56–59], their effects on pre-retinal NV as seen in vision-threatening proliferative diabetic retinopathy had not been explored. In addition, while multiple studies have evaluated the effect of CXCR2 activation on endothelial cells in other organ systems, its effects on hRMEC had not been investigated until now.

To address these knowledge gaps, we mimicked a physiologic paracrine stimulus to explore our hypothesis that CXCR2 ligand upregulation may connect chronic inflammation to retinal angiogenesis in DR pathophysiology. For this, we treated hRMEC with control vs. conditioned media from hMC stimulated with IL-1β and TNFα. This hMC-CM, which had elevated protein levels of IL-8 and CXCL1, but no detectable VEGF, enhanced hRMEC proliferation and migration. To confirm the role of IL-8 in inducing these phenotypes, we stimulated hRMEC directly with IL-8, likewise resulting in enhanced proliferation and migration. The effects of both direct and paracrine hRMEC stimulation (with IL-8 and hMC-CM, respectively) on proliferation and migration were ameliorated by CXCR2 inhibition.

Finally, we show that interfering with CXCR2 signaling either genetically or with systemic or intravitreal pharmacologic inhibition reduced pre-retinal NV in the OIR model, in which MC expression of the mouse IL-8 homologue, CXCL1, is known to coincide with NV onset and progression. Although NV is usually found to be proportional to the amount of avascular retina in OIR, CXCR2 inhibition reduced NV without detectable changes in the area of avascular retina, uncoupling these effects. This may be desirable for treating conditions such as retinopathy of prematurity, where treatments would ideally block pathologic NV without interfering with physiologic retinal vascular development.

Additionally, we found that the decrease in NV in the OIR model after CXCR2 inhibition was not dependent on changes in VEGF levels. Of the transcripts of interest, only *Il1b* was upregulated in p17-OIR *Cxcr2*^*-/-*^ mice as compared to WT. Given our hypothesis that IL-1β induces the expression of CXCL1, linking inflammation to angiogenesis, we suspect that the observed *Il1b* upregulation in *Cxcr2*^*-/-*^ mice may be the result of a feedback loop caused by the inhibition of CXCL1 signaling via CXCR2, preventing the neovascular response to hypoxia. Taken together, these data suggest that the activation of the chemokine receptor CXCR2 may be important in DR pathophysiology and that Müller cells may link inflammatory and angiogenic responses towards DR progression to vision-threatening stages, potentially through IL-1β induction of CXCR2 ligands.

Whether it is through VEGF-dependent or independent mechanisms, it is likely that CXCR2 activation by ligands such as IL-8 plays an important role in human angiogenic retinopathies. In humans with DR, the levels of IL-8 more strongly correlate with disease progression to vision-threatening DR stages than levels of VEGF, while vitreous levels of IL-8 inversely correlate with response to anti-VEGF therapy for DME [5, 7, 60–62]. Furthermore, polymorphisms in genes encoding members of both CXCL8:CXCR1/2 and the VEGF:VEGFR signaling axes are linked to morbidity and response to anti-VEGF therapy in angiogenic diseases, including AMD and cancers [9, 11, 14, 63–65]. Additionally, others have demonstrated that placental growth factor (PlGF), a member of the VEGF family also inhibited by the anti-VEGF drug, aflibercept, promotes the expression of CXCR2 ligands (i.e., IL-8, CXCL2) [66, 67]. This suggests that the effects of aflibercept may indirectly involve changes in CXCR2 activation. Furthermore, IL-8 levels have been shown to be elevated in AMD patients for whom anti-VEGF therapy fails [8]. Taken together, this suggests that targeting CXCR2 or its ligands may be beneficial for patients with retinal NV and those who fail anti-VEGF therapies. Future studies investigating potential interaction between CXCR2 and VEGFR2 signaling systems or other VEGF family intermediates may further illuminate DR mechanisms and inform development of novel therapies.

Several pharmaceutical companies have developed small-molecule and peptide-based inhibitors to selectively inhibit the IL-8 receptors CXCR1 and CXCR2 [24–26, 68, 69]. Of these, Reparixin, a small-molecule inhibitor from Dompe Pharmaceuticals, is designated by the FDA as an orphan drug for prevention of delayed graft dysfunction after organ transplantation [25, 69]. This drug is in multiple clinical trials for diseases ranging from breast cancer (phase 1 and 2) and ischemia–reperfusion injury after solid organ transplant (phase 2), to islet-cell transplantation in type 1 diabetes (phase 2 and 3). In addition, a related compound, ladarixin, which has equivalent selectivity for CXCR1 and CXCR2 and is orally bioavailable, can delay diabetes onset in non-obese diabetic mice and is currently in a phase 2 clinical trial for new-onset diabetes type 1 (NCT02814838, ClinicalTrials.gov). Unfortunately, these trials have yielded conflicting results, and our own experiments with *Cxcr2* KO mice have raised concerns that pleiotropism in signals from IL-8 receptors (CXCR1, CXCR2, ACKR1) and their multiple ligands (CXCL1, 2, 3, 5, 6, 7 and 8) may contribute to both protective and deleterious effects on health [30]. Therefore, before translation of this work into clinical practice, it will be paramount to evaluate the safety of chronic administration of IL-8 receptor inhibitors in humans.

In our study, CXCR2 inhibition decreased pre-retinal NV in OIR by 30–40% without significantly affecting VEGF levels, suggesting a VEGF-independent contribution of CXCR2 activation to retinal angiogenesis. Drugs blocking CXCR2 activation may thus be beneficial alone or in combination with anti-VEGF therapies, particularly for treatment-naïve patients with DR. However, given concerns for the safety of long-term inhibition of CXCR2 raised by mouse experiments, [30] exploring the effects of cell-specific inhibition of CXCR2 in endothelial, glial, and immune cells through conditional knockouts may help guide the development of cell-specific CXCR2 therapies that effectively treat disease while reducing deleterious off-target effects. Finally, exploring relationships between polymorphisms in genes encoding IL-8 receptors and DR development, progression, and response to therapy in biobanks may help inform future personalized strategies to guide prognosis and management for individuals with DR.

## Methods

### Primary human retinal microvascular endothelial cell (hRMEC) culture

hRMEC were purchased from Cell Systems (#ACBRI 181, Lot #181.01.03.01.02, Received Passage 3 on 09/05/2017, Expiration Date: 12/31/2026, Cell Systems; Kirkland, WA, USA) and cultured in attachment factor-coated (#4Z0-210, Cell Systems; Kirkland, WA, USA) T75 flasks in 10% FBS phenol red-free endothelial basal medium with SingleQuots (#CC-4133, Lonza; Walkersville, MD, USA) and streptomycin (hereunto referred to as hRMEC media). All cultures were maintained under 5% CO_2_ at 37 °C in a humidified incubator. Media was replaced the day after seeding, as well as every other day until use.

### hRMEC immunocytochemistry to confirm CD31, CXCR1, and CXCR2 expression

hRMEC were plated onto Falcon Chambered Cell Culture Slides (#08–774-26, Fisher Scientific; Tewksbury, MA, USA) at a density of 25,000 cells/well. Cells were grown to ~ 80% confluency and stimulated overnight with 25 ng/mL VEGF-A165 (#293-VE/CF, Bio-Techne; Minneapolis, MN, USA) and 1 ng/mL TNFα (#210-TA/CF, Bio-Techne; Minneapolis, MN, USA) as pro-inflammatory and pro-angiogenic stimuli. Each well was washed with DPBS and fixed in 4% paraformaldehyde diluted in DPBS. The cells were then washed with 1X Tris Buffered Saline (TBS) and permeabilized with 0.2% Triton X-100 (#T8787-100ML, Sigma-Aldrich; Saint Louis, MO, USA) diluted in TBS. Each well was washed again in TBS then blocked in 10% Normal Goat Serum (#50062Z, RRID: AB_2532938, Life Technologies Corporation; Frederick, MD, USA) containing 0.3% Triton X-100 at room temperature for 2 h. The cells were incubated overnight in an anti-CD31 FITC-conjugated antibody (Mouse, 1:500, #ab13466, RRID: AB_300373, Abcam Limited; Waltham, MA, USA) and either an anti-CXCR2 primary antibody (Rabbit, 1:250, #PA1-31217, RRID: AB_2126488, Invitrogen; Carlsbad, CA, USA) or an anti-CXCR1 primary antibody (Rabbit, 2 µg/mL, #711234, RRID: AB_2633138 Invitrogen; Carlsbad, CA, USA) in blocking solution. The cells were washed with 0.3% Triton X-100 in TBS for 2 h before the secondary antibody, Alexa Fluor Goat Anti-Rabbit 594 (1:1000 #A11012, RRID: AB_2534079, Invitrogen; Carlsbad, CA, USA) diluted in blocking solution, was added for 1 h. All wells were then washed in 0.3% Triton X-100 in TBS and incubated with NucBlue Live ReadyProbes Reagent (#R37605, Invitrogen; Carlsbad, CA, USA) for 20 min. The wells were removed from the slides, which were then coverslipped with ProLong Gold Antifade Mountant (#P36930, Invitrogen; Carlsbad, CA, USA). Slides were imaged at 10X using an Olympus FV-1000 inverted microscope.

### Primary human Müller cell (hMC) isolation and culture

hMC were isolated from retinas dissected from human donor eyecups (National Disease Research Interchange Philadelphia, PA, USA) and dissociated in Dulbecco’s Modified Eagles Medium (DMEM; #11885084, Life Technologies; Carlsbad, CA, USA) containing trypsin (#27250018, Life Technologies; Carlsbad, CA, USA) and collagenase (#C9722, Sigma-Aldrich; Saint Louis, MO, USA) following established methods [70]. The identity and purity of primary hMC were confirmed by immunocytochemistry with antibodies against cellular retinaldehyde-binding protein (#sc-59487, Santa Cruz Biotechnology; Dallas, TX, USA) and glial fibrillary acidic protein (#NB30014, RRID: AB_2060981 Fisher Scientific; Suwanee, GA, USA). Passages 4 to 6 were used for all experiments.

### Cytokine stimulation of hMC and conditioned media generation

hMC were cultured in T75 flasks with DMEM (#11885084, Life Technologies; Carlsbad, CA, USA) containing 10% FBS, penicillin, and streptomycin (hereunto referred to as hMC media) until reaching ~ 80% confluency and were then plated onto plastic VWR 6-well plates at a density of 30,000 cells/well in hMC media. Once they reached ~ 80% confluency, they were starved in DMEM containing 2% FBS, penicillin, and streptomycin for 12 h. The starvation media was then aspirated and the hMC washed twice with warm PBS. The hMC were then stimulated for 2 h in DMEM hMC media with either vehicle [0.1% bovine serum albumin (BSA; #A8806, Sigma-Aldrich; St. Louis, MO, USA) in phosphate buffered saline (PBS; #10010031, Thermo Fisher Scientific; Asheville, NC, USA)] or 1 ng/mL of both human recombinant IL-1β (#201-LB/CF, R&D Systems; Minneapolis, MN, USA) and TNFα (#210-TA, R&D Systems; Minneapolis, MN, USA), which were reconstituted in vehicle. After 2 h, media was aspirated and the hMC washed once with warm PBS. Then, 900µL of 2% FBS EBM without SingleQuots was added to the hMC to be conditioned by the secretions of the hMC for a period of 6 h. After 6 h, this hMC-CM was harvested (see Fig. 1a) and immediately used for proliferation or scratch assays. Biological triplicates were immediately aliquoted and stored at − 80 °C for ELISA.

### ELISAs of conditioned media

ProQuantum Human IL-8 (#A35575, Thermo Fisher Scientific; Waltham, MA, USA), Human VEGF-A (#A35602, Thermo Fisher Scientific; Waltham, MA, USA), and Human CXCL1 (#A42896, Thermo Fisher Scientific; Waltham, MA, USA) Immunoassay Kits were used for enzyme-linked immunoassays of 3 harvested hMC-CM biological replicates. Samples and standards of 5µL were prepared with 3 technical replicates and diluted ten-fold in accordance with the manufacturer’s instructions. PCR amplification of samples and standards was then performed using an Applied Biosystems Step OnePlus Real-Time PCR System (#4376600, Thermo Fisher Scientific; Waltham, MA, USA).

### Stimulation of hRMEC

hRMEC were either stimulated with (1) hMC-CM or its unconditioned EBM control or (2) human recombinant IL-8 or its BSA control. Human IL-8 (77aa) (#200–08, PeproTech; Cranbury, NJ, USA), which was reconstituted in 0.1% BSA (#A8806, Sigma-Aldrich; St. Louis, MO, USA), was diluted in PBS to 25 ng/mL, and an equivalent volume of 0.1% BSA vehicle was diluted identically in PBS for an effective control concentration of 0.0000025% BSA in PBS. hRMEC proliferation and migration were then monitored in the presence and absence of CXCR2 inhibitors or their DMSO vehicles.

### Proliferation assays

hRMEC were seeded onto Agilent 96-well Microplates (#204626-100, Agilent; Santa Clara, CA, USA) at a density of 3,500 cells/well in hRMEC media. After adherence, the hRMEC were starved in EBM containing 2% FBS for 8 h before adding conditioned media (hMC-CM or unconditioned EBM with either 50 nM SB and 75 nM or 0.000381% DMSO) or direct stimulus (25 ng/mL IL-8 or 25 ng/mL BSA with either 50 nM SB or 0.0000587% DMSO) treatments. The plate was then transferred to a BioTek Cytation 5 Cell Imaging Multimode Reader (Agilent; Santa Clara, CA, USA) set to a temperature of 37 °C and supplied with 5.0% CO_2_. Images were taken every 2 h for 36 h, and cell count was quantified using Gen5 software (Version 3.11.19). All cell counts were normalized to their treatment group directly after the addition of treatment and evaluated with an area under the curve analysis.

### Scratch assays

hRMEC were grown to ~ 80% confluency in T75 flasks and then plated onto Corning 96-well Microplates (#3598, Corning Incorporated; Corning, NY, USA) in 0.2 mL hRMEC media at a density of 33,000 cells/well. Once the cells reached ~ 90% confluency, they were pre-treated with 50 nM SB + 75 nM Rx or 0.000381% DMSO for 7 h in the hMC-CM scratch assays or with 50 nM SB or 0.0000587% DMSO for 4–5 h in the IL-8 direct-stimulation scratch assays. The Agilent BioTek AutoScratch tool (AUTOSCRATCH, Agilent; Santa Clara, CA, USA) was then used to make uniformly sized wounds in each well. Each well was then washed twice with warm PBS using a multi-channel pipette to remove non-adherent cells without disrupting the cell monolayers. For the hMC-CM scratch assay, hRMEC were then stimulated with hMC-CM or unconditioned EBM control with either 50 nM SB and 75 nM Rx or 0.000381% DMSO. For the IL-8 direct stimulation scratch assays, hRMEC were treated with 25 ng/mL IL-8 or its BSA control with either 50 nM SB or 0.0000587% DMSO. The plate was then transferred to the BioTek Cytation 5 Cell Imaging Multimode Reader and cultured at 37 °C and 5.0% CO_2_. Confluency was determined by the BioTek Scratch Assay App (Version 1.03). For assays of direct IL-8 stimulation, data from 2 consecutive scratch assays was combined prior to analysis. Confluency was multiplied by a constant wound factor *F* such that a fully-confluent well corresponded to a confluency of 100% (*F* = 3.3 for the hMC-CM migration, Fig. 4; *F* = 2.8 for the IL-8 direct stimulus migration, Fig. 6). For the analysis of hMC-CM stimulation of hRMEC migration, Fig. 4, n = 4 for Control + DMSO and hMC-CM + SB/Rx groups, and n = 3 for Control + SB/Rx and hMC-CM + DMSO groups. For the analysis of IL-8 direct stimulus of hRMEC migrations, Fig. 6, n = 11 for BSA + DMSO, BSA + SB, and IL-8 + DMSO groups, and n = 9 for the IL-8 + SB group. Wells were excluded from analysis if they possessed non-uniform scratches, inadequate confluency, or in the event of autofocus malfunction. Data was then evaluated with an area under the curve analysis.

### TUNEL assay

hRMEC were grown to ~ 80% confluency in a T75 flask and then plated onto Corning 96-well Microplates at a density of 3000 cells/well. Cells were then treated with 0.0000587% DMSO, 50 nM SB, or 2% FBS in EBM and allowed to grow for 36 h prior to being washed once with PBS, fixed at room temperature with 4% paraformaldehyde (#15710, Electron Microscopy Sciences; Hatfield, PA, USA) in PBS, permeabilized with 0.25% Triton X-100 (#T8787-100ML, Sigma-Aldrich, St. Louis, MO, USA), and then washed twice with deionized water. The positive control group was then incubated with a DNase cocktail for 30 min according to the instructions of the Click-iT™ PLUS TUNEL Assay Kit (#C10617, Invitrogen; Carlsbad, CA, USA) and then washed twice with deionized water. All wells were then incubated with TdT Reaction buffer at 37 °C. Each well was then washed twice with 3% BSA in PBS before incubation with Click-iT™ Plus TUNEL reaction cocktail at 37 °C while protected from light. Wells were then washed with 3% BSA in PBS and imaged using an Olympus FV-1000 inverted microscope (Olympus Corporation; Tokyo, JPN). Two random, non-overlapping regions of interest were imaged and analyzed for each treatment well.

### Animals

All animal studies were conducted in accordance with the Guide for the Care and Use of Laboratory Animals [71] in a protocol approved by the IACUC at Vanderbilt University Medical Center (VUMC). *Cxcr2* knockout (*Cxcr2*^**-/-**^) and wild-type littermate mice were obtained by breeding heterozygous B6, 129S(C)-*Cxcr2*^tm1Mwm^/J from Jax (RRID: IMSR_JAX:006848) mice [72]. C57BL/6 mice, purchased from Jax (RRID: IMSR_JAX:000664), were periodically used to refresh the heterozygous *Cxcr2*^±^ colony. Mice were housed in autoclaved cages containing paper bedding in level 5A housing conditions at VUMC. Mice were provided water, fed ad-lib with autoclaved pellets (5L0D, PicoLab® Laboratory Rodent Diet, LabDiet; Richmond, Indiana, USA), and were supplemented with 2 oz of 76A DietGel (72-07-5022, Clear H20; Westbrook, ME, USA) twice per week. Observers masked to animal genotype, experimental group, and diet performed all data acquisition and analyses described.

### Genotyping

Sigma Red Extract-N-Amp Tissue PCR Kit (#XNAT2-1KT, Sigma-Aldrich; Saint Louis, MO, USA) was used to extract and amplify genomic DNA in tail tissue collected from littermate pups for genotyping. Samples were incubated with DNA isolation buffer at room temperature and then at 95 °C on a heat block. PCR amplification was conducted using WT forward 16,927 (5′-AGA ATT GGG GTC AGG GTA’A-3′), WT reverse 16,928 (5′-CCC TTA GGC TGC AAA TGA’C-3′), mutant forward oIMR6916(5′-CTT GGG TGG AGA GGC TAT’C-3′), and mutant reverse oIMR6917 (5′-AGG TGA GAT GAC AGG AGA’C-3′) primers. Samples were programmed in a MiniAmp Thermal Cycler (ThermoFisher Scientific; Waltham, MA, USA) as follows: 94 °C for 3 min, 30 cycles of 94 °C for 1 min, 62 °C for 1 min, 72 °C for 1 min, 72 °C for 10 min, and then held at 4 °C. Gel electrophoresis was performed with parameters of 100 V, 0.08 A, and 8 W for 1:15 h. Gel was prepared by adding 1.5 g of agarose (#A9539-100G, Sigma-Aldrich; Saint Louis, MO, USA) to 100 mL IX TAE buffer (#46–010-CM, Corning Incorporated; Corning, NY, USA) diluted in PBS, heating the mixture in the microwave to fully dissolve the agarose. SYBR safe (#S33102, Invitrogen; Carlsbad, CA, USA) was added for staining and the solution allowed to polymerize at room temperature. DNA fragment size was found at 214 bp for WT and at 280 bp for *Cxcr2* mutant.

### Oxygen-induced retinopathy (OIR)

Newborn mice and their nursing mothers were placed in a plexiglass chamber equipped with an oxygen control system (OxyCycler Model A84; BioSpherix, Parish, NY, USA) from postnatal day 7 (p7) to postnatal day 12 (p12). During this time, the oxygen concentration to which the mice were exposed was maintained around 75% O_2_. At p12 (p12-OIR), mice were removed from the chamber and exposed to normoxic conditions (~ 21% O_2_) until p17. At p17 (p17-OIR), mice were euthanized and their eyes enucleated as described in Enucleation, Fixation, and Retinal Dissection. Some retinas were excluded from analysis due to sample processing error.

For OIR with *Cxcr2*^*-/-*^ vs. WT mice, six sets of mixed-genotype litters were subjected to OIR. Only litters containing at least one live *Cxcr2*^*-/-*^ (KO) and one live *Cxcr2* wild-type mouse were included in analysis.

For OIR with daily intraperitoneal (IP) injections of SB225002 or DMSO Vehicle, mice were removed from the hyperoxic chamber at p12-OIR and administered daily intraperitoneal (IP) injections of either 4 mg/kg SB225002 or 0.008% DMSO from p12-p17 using U-100 insulin syringes (#26026, Comfort Point; Fort Worth, TX, USA).

For OIR with intravitreal (IVIT) injections of SB225002 or DMSO Vehicle, solutions of 1.5 µM SB or 0.0017605% DMSO were prepared by diluting stock supply concentrations in sterile PBS. 1µL of the desired treatment was injected into the vitreous of anesthetized p12 mice as described in Intravitreal Injection.

### Intravitreal injection

After removal from the OIR OxyCycler chamber, p12 pups were treated topically with 1% tropicamide (#17478-102, Arkorn; Gurnee, IL, USA) and 0.5% proparacaine hydrochloride (#17478-263, Arkorn; Gurnee, IL, USA) and then anesthetized with inhalant isoflurane. Eyes were injected with 0.5 µL 0.0017605% DMSO vehicle or 1.5 µM SB225002 (SB, #13336, Cayman Chemical; Ann Arbor, MI, USA) in the contralateral eye to control for intrinsic differences between animals and for any technical biases in injection administration. Eyes were treated with 0.3% tobramycin antibiotic (#61314–643, Sandoz; Princeton, NJ, USA) immediately after each injection. Mice were allowed to recover with their nursing dam in room air until p17-OIR, the target age for retinal analysis.

### Enucleation, fixation, and retinal dissection

Following OIR, after euthanasia, mouse eyes (mostly at p17-OIR) were enucleated, fixed for 2 h in 4% paraformaldehyde diluted in PBS, and washed three times with 1X PBS. Several naïve, uninjected eyes were also enucleated at p12-OIR to evaluate the avascular area prior to the neovascular phase of OIR. Retinas used for IHC were micro-dissected from the globes in an Olympus SZ61 Zoom Stereomicroscope and kept in PBS before immunohistochemistry.

### Immunohistochemistry

Micro-dissected retinas from fixed eyes were blocked with 10% Normal Goat Serum (#50062Z, RRID: AB_2532938, Life Technologies; Frederick, MD, USA) in PBS containing 0.3% Triton X-100 (#T8787-100ML, Sigma-Aldrich; St. Louis, MO, USA) for 2 h at room temperature in the wells of Agilent 96-well Microplates. They were then incubated overnight at 4 °C with an antibody against glial fibrillary acidic protein (GFAP) (Rabbit, 1:500, #Z0334, DAKO; Santa Clara, CA, USA). The retinas were then washed with TBS containing 0.3% Triton X-100 (TBS-T) before they were incubated with 1:75 Alexa Fluor 488-Isolectin B4 (IB4) conjugate antibody (#I21411, Invitrogen; Carlsbad, CA, USA) and 1:1000 Gt anti Rabbit Alexa Fluor 594 (#A11012, Invitrogen; Carlsbad, CA, USA) in blocking solution. The retinas were then washed again with TBS-T. Any residual vitreous was then removed from the retinas and four radial cuts were made before flat mounting the retinas onto glass microscope slides inProLong Gold Antifade Mountant (#P36930, Invitrogen; Carlsbad, CA, USA). After drying overnight in RT, the flat mounts were montage imaged at 4X using an Olympus FV-1000 inverted microscope.

### Avascular area and neovascular area analyses

Avascular (AVA), neovascular (NVA), and total (TRA) retinal areas were traced by a masked investigator using Adobe Photoshop (Version 26.1.0; San Jose, CA, USA). Area measurements, represented by the number of pixels in each selection, were obtained using Adobe Photoshop’s “Histogram” function. Traced AVA, NVA, and TRA measurements were then averaged for each litter’s experimental and control group and analyzed with paired t-tests to account for intrinsic differences between litters*.*

### Quantitative real-time PCR (qRT-PCR) of OIR and RA mouse retinas

Four mixed-genotype litters were subjected to 75% O_2_ from p7 to p12 and enucleated on p17. Following OIR, mouse eyes were dissected immediately, and the retinas were placed into 1% β-mercaptoethanol in Buffer RLT. The same procedure was carried out for 2 litters of room air (RA) p17 and p18 WT mice not subjected to OIR. Samples were homogenized with the Qiagen TissueLyser LT (#85600, Qiagen; Hilden, Germany) before centrifugation, with the supernatant then extracted for RNA isolation using the RNeasy Mini Kit (#74106, Qiagen; Hilden, Germany). RNA concentration was quantified with the BioTek Synergy Mx Microplate Reader (Aligent; Santa Clara, CA, USA) for cDNA synthesis calculations. 

RNA samples were reverse-transcribed to cDNA with the Applied Biosystems High Capacity cDNA Reverse Transcription Kit (#4368813, Thermo Fisher Scientific; Waltham, MA, USA) and placed into the Applied Biosystems MiniAmp Plus Thermal Cycler (#A37835, Thermo Fisher Scientific; Waltham, MA, USA). qRT-PCR was conducted using a TaqMan gene expression assay and separate solutions of TaqMan Universal Master Mix (#4304437, Thermo Fisher Scientific; Waltham, MA, USA), RNase free water, and the following Thermo Fisher Scientific gene expression primers: *Tbp* (Mm01277042_m1), *Kdr* (Mm01222421_m1), *Cxcr1* (Mm00731329_s1), *Il1b* (Mm00434228_m1), *Vegf* (Mm00437306_m1), *Cxcr2* (Mm99999117_s1), *Tnf* (Mm00443258_m1), and *Cxcl1* (Mm04207460_m1). Each primer master mix was combined with 2 technical replicates of each cDNA sample in a MicroAmp Optical 96-Well Reaction Plate (#4306737, Thermo Fisher Scientific; Waltham, MA, USA) sealed with MicroAmp Optical Adhesive Film (#4311971, Thermo Fisher Scientific; Waltham, MA, USA). The Applied Biosystems QuantStudio3 Real Time PCR Instrument (Thermo Fisher Scientific; Waltham, MA, USA) amplified the cDNA under the following conditions: an initial hold stage of 50 °C for 2 min and 95 °C for 10 min, followed by 40 cycles of 95 °C for 15 s and 60 °C for 1 min. All ΔC_T_ values were calculated relative to the technical replicate average of TBP. For OIR *Cxcr2*^*-/-*^ vs. WT results, all fold change values were calculated relative to the WT ΔC_T_ averages of each primer for each litter individually. For the RA WT vs. OIR WT results, fold change values were calculated relative to the RA WT ΔC_T_ averages of each primer, irrespective of litter.

### Statistics

Graphs and statistical analyses were performed using GraphPad Prism version 10.4.1 (GraphPad Software, CA, USA). We first identified and removed outliers in each data set via Grubb’s test (α = 0.001). Subsequently, we used D’Agostino-Pearson tests, or Shapiro–Wilk tests for smaller datasets, to determine the normality of each distribution. For normally distributed datasets, we performed parametric statistics (t-tests or ordinary one-way ANOVA). Paired t-tests were used for OIR statistical comparisons to control for innate variations between litters. Welch’s t-tests were performed since many sample sizes were uneven, and the standard deviations between groups were not assumed to be equivalent. For non-normally distributed data, which only occurred for single-comparison analyses, we carried out non-parametric statistics (Mann–Whitney or Wilcoxon tests). Multiple comparisons were accounted for by applying the Šídák correction. We defined statistical significance as a *p*-value of 0.05 or less. The data in results is presented as mean ± standard deviation with specific *p*-values.

## Data Availability

Data is publicly available within this manuscript.
